# Investigating the Efficacy of EGFR-TKIs and Anti-VEGFR Combination in Advanced Non-Small Cell Lung Cancer: A Meta-Analysis

**DOI:** 10.3390/cancers16061188

**Published:** 2024-03-18

**Authors:** Prashant Sakharkar, Sonali Kurup, Subrata Deb, Kaitlin Assaad, Dayna Gesinski, Erysa J. Gayle

**Affiliations:** 1 College of Pharmacy, Larkin University, Miami, FL 33169, USA; sdeb@alumni.ubc.ca; 2 College of Pharmacy, Ferris State University, Big Rapids, MI 49307, USA; sonalikurup@ferris.edu (S.K.);; 3 College of Biomedical Sciences, Larkin University, Miami, FL 33169, USA

**Keywords:** NSCLC, non-small cell lung cancer, epidermal growth factor receptor, tyrosine kinase inhibitors, EGFR-TKIs, epidermal growth factor receptor tyrosine kinase inhibitors, anti-VEGFR, vascular endothelial epidermal growth factor receptor, erlotinib, bevacizumab

## Abstract

**Simple Summary:**

Combining certain drugs that target specific proteins in cancer cells has been found to help people with advanced non-small cell lung cancer live longer. We systematically reviewed several clinical trials and synthesized evidence to see how well the epidermal growth factor receptor tyrosine kinase inhibitors (EGFR-TKIs) and anti-vascular endothelial growth factor receptor (anti-VEGFR) combination treatment works. These drugs target specific proteins involved in cancer growth. We found that when EGFR-TKI is used together with another drug that blocks blood vessel growth (anti-VEGFR), they can delay the cancer from getting worse, but they do not necessarily make people live longer overall. This seems to be true irrespective of whether the treatment is used as the first option or later, and whether it’s an older or newer type of the EGFR-TKI drug.

**Abstract:**

Introduction: The epidermal growth factor receptor tyrosine kinase inhibitors (EGFR-TKIs) in combination with anti-vascular endothelial growth factor receptor (VEGFR) agents have shown improved survival outcomes in recent studies. However, its efficacy related to survival outcomes as a first- or second-line agent and based on generations remains to be explored. This study estimated the survival outcomes of EGFR-TKIs plus anti-VEGFR in combination in defined populations of advanced non-small cell lung cancer (NSCLC) patients overall, as a first- or second line of treatment, with different generations of EGFR-TKIs and EGFR-TKIs plus bevacizumab combination as a subgroup. Methods: A literature search was conducted using PubMed, SCOPUS, Cochrane Library, and ClinicalTrials.gov databases through June 2023 to identify primary research reporting the survival outcomes of EGFR-TKIs in combination with anti-VEGFR agents in patients with advanced NSCLC. Studies that were single-arm, published in non-English languages, and had missing data on survival outcomes were excluded. A meta-analysis was conducted to generate pooled hazard ratios (HRs) with 95% confidence intervals (CI) for overall survival (OS) and progression-free survival (PFS). Methodological quality and risk of bias in studies were assessed using the Cochrane Handbook for Systematic Reviews of Interventions risk of bias tool. Results: A total of 20 randomized controlled trials were included in the qualitative synthesis, and 11 (2182 participants) were included in the meta-analysis. Patients’ median age ranged from 58 to 68 years; 36% to 70% of patients were female; most of them had IIIa/b to IV stage cancer. In meta-analyses, the EGFR-TKIs plus anti-VEGFR combination resulted in improved PFS (HR, 0.73; 95% CI: 0.61, 0.86; *p* < 0.00001) in patients with advanced NSCLC but had no impact on OS (HR, 0.93; 95% CI: 0.79, 1.10; *p* = 0.41). The first line of treatment and first-generation EGFR-TKIs with the combination also improved the PFS (HR, 0.64; 95% CI: 0.57, 0.71; *p* < 0.00001; HR, 0.63; 95% CI: 0.56, 0.71; *p* < 0.00001) respectively, however, had no impact on OS. Conclusions: Our meta-analysis indicated EGFR-TKIs with anti-VEGFR in combination not only improved overall PFS but also showed similar results to a first line and first-generation agent compared to EGFR-TKI alone.

## 1. Introduction

Lung cancer is a leading cause of cancer-related mortality in the United States, with approximately 85% of patients having non-small cell lung cancer (NSCLC) [1]. Unfortunately, about 75% of NSCLC patients are diagnosed at an advanced stage, leading to a poor prognosis despite the availability of new diagnostic and therapeutic options [2]. The 5-year survival rate for these patients is less than 20% [1]. In patients with epidermal growth factor receptor (EGFR) mutation-positive lung cancer, EGFR tyrosine kinase inhibitors (TKIs) are considered the standard first-line treatment [3,4]. However, acquired resistance to first-generation EGFR TKIs limits the median progression-free survival (PFS) to approximately one year [5,6,7]. One possible approach to overcome acquired resistance and improve outcomes is to combine EGFR-TKIs with other drugs. EGFR-TKIs are targeted therapies that belong to one of three generations. The first-generation EGFR-TKIs, such as erlotinib and gefitinib, the second-generation, including dacomitinib and afatinib, and the third-generation agent, Osimertinib, are approved as first-line agents for NSCLC harboring EGFR mutations [3,4]. Despite their efficacy, EGFR-TKIs are associated with adverse effects, including rash, diarrhea, nausea and vomiting, anemia, and fatigue, with skin rash and interstitial lung disease being the most common [8].

Vascular endothelial growth factor (VEGF) and its related receptor subtypes (VEGFR1, VEGFR2, and VEGFR3) are the major drivers of angiogenesis in NSCLC [9]. Anti-VEGF agents can be either antibodies that neutralize VEGF protein (bevacizumab) or VEGFR2 (ramucirumab) or small molecules that inhibit VEGFR-related tyrosine kinase enzymes (e.g., apatinib) [10]. Bevacizumab is a recombinant antiangiogenic monoclonal antibody that targets the VEGF signaling pathway, inhibiting tumor angiogenesis and suppressing growth. Combination therapy with EGFR-TKIs (e.g., erlotinib) and anti-VEGFR (e.g., bevacizumab) agents has been shown to prolong PFS in patients with NSCLC in a few randomized controlled trials [11]. The combination of bevacizumab and erlotinib targets different tumor growth pathways, thus potentially complementing each other’s mechanisms to control tumor growth. A phase II trial by Roselle and colleagues was the first to demonstrate the efficacy of this combination in the EGFR-mutant subgroup, and several other randomized controlled trials (RCTs) have shown the prolongation of PFS and objective response rate (ORR) in advanced NSCLC with erlotinib plus bevacizumab [12]. However, the effects of this combination in advanced mutation-positive (EGFRm+) NSCLC patients remain inconclusive. Multi-targeted ATP-competitive VEGFR inhibitors have also been investigated, including apatinib, anlotinib, vandetanib, sunitinib, and sorafenib [9]. The combination of erlotinib and ramucirumab, and erlotinib and bevacizumab, has been approved for the first-line treatment of EGFR-positive advanced NSCLC patients [13].

The results of the meta-analysis by Deng and colleagues [14] showed that the combination of EGFR-TKIs and anti-VEGFR agents, specifically erlotinib plus bevacizumab, was associated with a significant improvement in PFS compared to EGFR-TKI alone. However, no significant difference was observed in overall survival (OS) between the two treatment groups. Subgroup analyses also showed that combination therapy as a first-line treatment was associated with a longer PFS compared to second-line therapy [14]. These findings suggest that the combination of EGFR-TKIs and anti-VEGFR agents may be a promising treatment strategy for patients with advanced NSCLC [13,15]. However, the efficacy of these combination therapies in terms of OS, as well as the first or second line of treatment, remains inconclusive. Further research is needed to identify optimal treatment strategies for NSCLC patients and improve their clinical outcomes. By utilizing a PICO framework, the Patient (NSCLC), Intervention (EGFR-TKIs plus Anti-VEGFR), Comparator (EGFR-TKIs), and Outcomes (PFS, OS, ORR), we conducted a meta-analysis to investigate whether the combination of EGFR-TKIs plus anti-VEGFR is associated with improved OS, PFS, and ORR compared to EGFR-TKIs alone in defined populations of advanced NSCLC patients. Additionally, we also assessed the efficacy of this combination as a first or second line and improvement in survival outcomes with different generations of EGFR-TKIs and EGFR-TKIs plus bevacizumab combination in subgroup analyses.

## 2. Materials and Methods

### 2.1. Literature Search

A literature search was conducted in accordance with the Preferred Reporting Items for Systematic Reviews and Meta-Analysis (PRISMA) Statement [16]. The protocol has been registered with Open Science Framework (OSF) registries (https://osf.io/p8zjc (accessed on 4 October 2023)). We searched PubMed, SCOPUS, Cochrane Library, and ClinicalTrials.gov using relevant and related keywords and MeSH terms: “EGFR”, “epidermal growth factor receptor”, “tyrosine kinase inhibitor”, “VEGFR”, “Anti-VEGFR”, “vascular endothelial growth factor receptor”, “vascular endothelial growth factor receptor tyrosine kinase inhibitors”, “VEGFR-TKIs” and “non-small cell lung cancer” through 2023. The final search of databases was performed in June 2023. Furthermore, we hand-searched references of relevant articles to retrieve additional publications.

### 2.2. Selection Criteria

We included studies that met the following criteria: (a) the trial design was phase II or phase III RCTs comparing an EGFR-TKI (erlotinib, gefitinib, afatinib, and osimertinib) to a combination of EGFR-TKI with an anti-VEGFR agent (bevacizumab, ramucirumab, apatinib) in patients with advanced NSCLC; (b) studies in patients with a confirmed diagnosis of advanced NSCLC; (c) only combination agents with a targeted mechanism of action were included; and (d) studies that reported survival related outcomes such PFS, OS, and ORR. Studies published in non-English languages, reviews, systematic reviews and meta-analyses, animal studies, and single-arm studies examined non-targeted therapy as a combination, and studies that did not report survival outcome measures such as PFS and OS were excluded. If multiple studies covered the same study population, relevant data from both studies were used, and the study with the most recent treatment outcome data was utilized.

### 2.3. Data Extraction

Studies included were reviewed by two independent investigators (S.K. and P.S.) in the title/abstract screening and final selection phase. Discrepancies were resolved by reaching a consensus. A structured data abstraction form was used to extract the following data: Author(s), year of publication, region of the study, type of study, trial phase, number of patients in both arms, median age, gender, race/ethnicity, mutation status (activating/resistant/undefined), therapeutic regimen and combinations, line of treatment (first/second) and survival outcomes. Data on adverse events (AE), including grade 3 and higher, hypertension, rash, diarrhea, and proteinuria, were also extracted.

### 2.4. Assessment of the Risk of Bias

The risk of bias was assessed using a tool described in the Cochrane Handbook for Systematic Reviews of Interventions. Two investigators independently assessed each trial based on random sequence generation, allocation concealment, blinding of participants, blinding of the outcome, incomplete outcome data, selective reporting, and other biases [17]. Discrepancies between investigators were resolved through the discussion.

### 2.5. Statistical Analysis

Our meta-analysis included PFS, OS, and ORR as the endpoints. The hazard ratio (HR) was used as a measure of the prognostic value. The pooled HR for PFS and OS with its 95% confidence intervals (CIs) were used to measure the treatment outcome. Heterogeneity was assessed by the χ^2^ test and expressed by the I^2^ index. The I^2^ values of <25%, 25–50%, and >50% were defined as low, mild, and substantial heterogeneity, respectively [18]. If the I^2^ value was <50% and *p* > 0.05, a fixed-effects model was used; otherwise, if the I^2^ value was ≥50% and *p* ≤ 0.05, a random-effects model was used. Sensitivity analysis was not performed as more than half of the eligible studies were open-label. Subgroup analyses were performed using a line of treatment and generations of EGFR-TKIs. An additional analysis was performed comparing EGFR-TKIs plus bevacizumab in combination versus EGFR-TKIs alone since most of the studies included had this combination, and it is most common in clinical practice. Publication bias was evaluated according to the funnel plot and Begg’s and Egger’s tests. All analyses were conducted using SPSS Ver. 28 (IBM Corp, Armonk, NY, USA)/STATA Ver. 14.0 (StataCorp, College Station, TX, USA) and Review Manager Ver. 5.4.1 (The Cochrane Collaboration, 2020). 

## 3. Results

### 3.1. Results of the Literature Search

**Figure 1:**
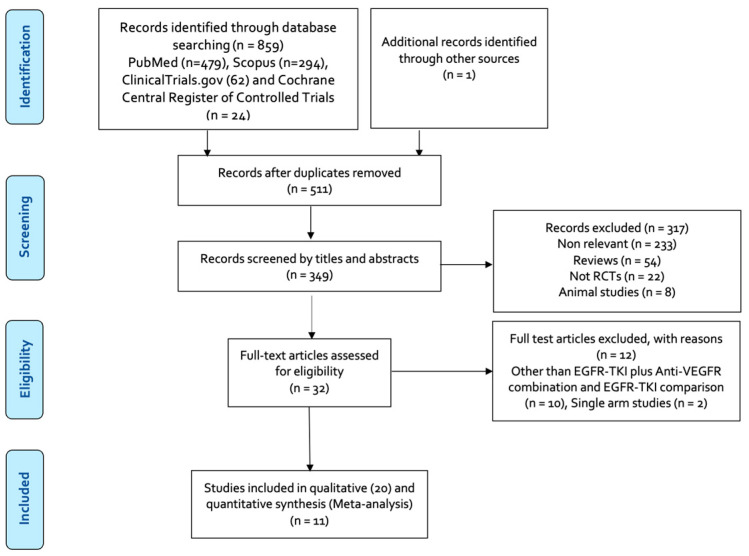
Flowchart of search and the eligible studies included in this meta-analysis.

Our review was conducted in accordance with the PRISMA Statement (Supplementary File S2). Our search identified a total of 860 publications, of which 511 were duplicates. A total of 20 studies that met our inclusion criteria were included in the qualitative synthesis, whereas 11 studies of defined patient populations with oncogenic drivers were included in meta-analyses (Figure 1).

### 3.2. Characteristics of the Included Studies

The demographic and summary of characteristics of the included studies are presented in Table 1 and Supplementary Table S3. Out of the 20 eligible trials [19,20,21,22,23,24,25,26,27,28,29,30,31,32,33,34,35,36,37,38], NEJ026 [34,35] and JO25567 trial [22,23] had dual publications. Twelve studies included first-generation EGFR-TKI agent erlotinib (15 studies) and gefitinib (two studies); three studies included third-generation EGFR-TKI and osimertinib. None of the studies had second-generation EGFR-TKIs. Twelve studies included VEGFR-targeted monoclonal antibodies, of which bevacizumab was included in ten studies; apatinib, pazopanib, sorafenib, and ramucirumab were included in one study each. Of the VEGFR-TKIs, two studies included vandetanib, a dual-targeted EGFR and VEGFR inhibitor. An equal number of studies, seven each, were conducted in Asia and globally. Nine of the studies included patients with activating mutations (del 19 EGFR or L858R EGFR), two with T790M EGFR, and seven studies included patients with undefined mutation status. A total of 951 patients had exon 19 deletion, 797 patients had exon 21 Leu858 Arg, and 236 had T790M mutation. Half of the studies included EGFR-TKI and anti-VEGFR combinations as the first and second line of treatment. Of these, patients with prior treatment with EGFR-TKI therapy were included in two studies, whereas seven studies had patients with undefined mutation status who were previously treated with cytotoxic agents. 

The number of patients included in these studies ranged from 81 to 1240. The median age of patients was between 58–68 years, and 36–70% of patients were female. The lowest proportion of patients with Asian race/ethnicity was 2% in these studies. The majority (14) of studies had patients with IIIa/b to IV stage cancer, three had stage IV, and one had stage I to IV cancer patients.

### 3.3. Risk of Bias and Quality Assessment

**Figure 2:**
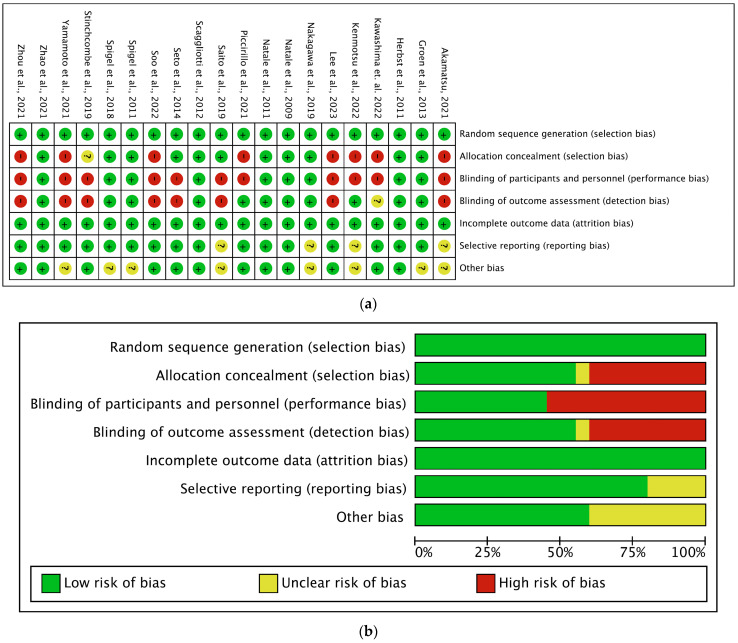
(**a**) Graphical representation of the risk of bias assessment [19,20,21,22,23,24,25,26,27,28,29,30,31,32,33,34,35,36,37,38]. (**b**) Summary of the risk of bias assessment.

The methodological quality of these studies and the risk of bias are presented in Figure 2a. All studies showed adequate random sequence generation; 11 publications indicated adequate allocation concealment [17,18]. Most publications showed low selective reporting. All studies were free of incomplete outcome data. Twelve publications guaranteed no other bias [17,18]. There was sufficient evidence to assess whether all studies were moderate or high quality (Figure 2a,b). Slightly less than half of the studies included were double/triple-blinded RCTs.

### 3.4. Meta-Analysis of Survival Outcome

#### 3.4.1. Progression-Free Survival

A total of eleven trials with defined oncogenic drivers were included in the meta-analysis. Studies assessing PFS [19,20,21,23,24,27,29,32,33,34,38] included 2182 patients (1091 each in combination and in monotherapy group) following EGFR-TKIs plus anti-VEGFR combination group compared to EGFR-TKI alone. PFS as a first- and second-line treatment was assessed in 1946 and 236 patients, respectively. The majority (7) of the studies were conducted in Asia. The median age of the population in these studies ranged between 58 to 68 years, and patients who used combination as a second-line treatment were a little older (67–68 years). The majority (nine) had a patient population with L858R or del19 EGFR mutation, where combination therapy was used as a first line of treatment.

**Figure 3:**
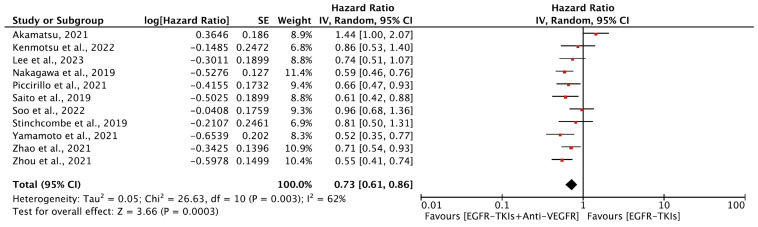
Meta-analysis showing the change in PFS between EGFR-TKIs + Anti-VEGFR and EGFR-TKIs: random-effects model [19,20,21,23,24,27,29,32,33,34,38]. PFS: Progression Free survival; EGFR-TKIs = Epidermal Growth Factor Receptor Tyrosine Kinase Inhibitors; Anti-VEGFR: Anti-Vascular Endothelial Growth Factor Receptor; SE: Standard Error; df: degrees of freedom; CI: Confidence Interval.

**Figure 4:**
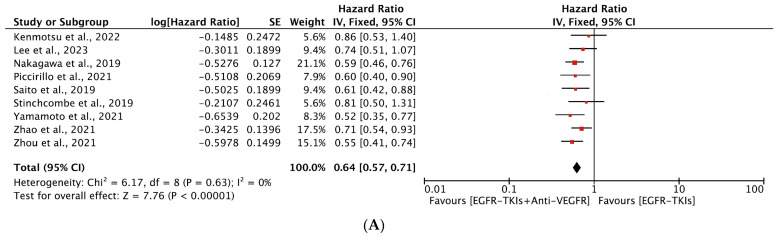

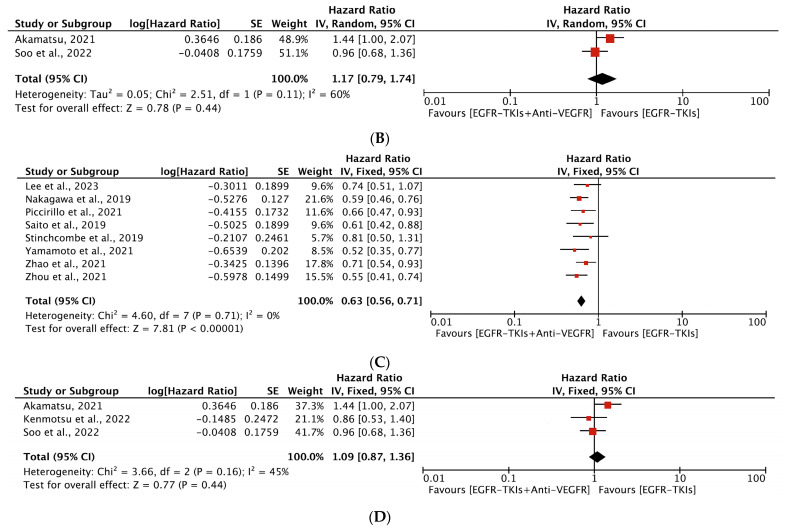
(**A**) Meta-analysis of subgroups showing change in PFS as a first line of treatment between EGFR-TKIs + Anti-VEGFR and EGFR-TKIs: fixed-effects model [19,20,21,23,24,29,32,33,34]. (**B**) Meta-analysis of subgroups showing the change in PFS as a second line of treatment between EGFR-TKIs + Anti-VEGFR and EGFR-TKIs: random-effects model [27,38]. (**C**) Meta-analysis of subgroups showing the change in PFS as a first generation (erlotinib and gefitinib) of EGFR-TKIs + Anti-VEGFR and EGFR-TKIs: fixed-effects model [19,20,21,23,24,29,32,34]. (**D**) Meta-analysis of subgroups showing the change in PFS as a third generation (osimertinib) of EGFR-TKIs + Anti-VEGFR and EGFR-TKIs: fixed-effects model [27,33,38]. PFS: Progression Free survival; EGFR-TKIs = Epidermal Growth Factor Receptor Tyrosine Kinase Inhibitors; Anti-VEGFR: Anti-Vascular Endothelial Growth Factor Receptor; SE: Standard Error; df: degrees of freedom; CI: Confidence Interval.

Six of the included studies reported statistically significant improvement in PFS. The overall median PFS ranged between 9.4 to 22.1 months for combination therapy and 9.6 to 20.2 months for monotherapy. The median PFS for combination as a first-line agent ranged from 13.7 to 22.1 months compared to 9.6 to 20.2 months for EGFR-TKI monotherapy. In comparison, median PFS was 9.4 to 15.4 months and 12.3 to 13.5 months as a second-line agent for the combination and monotherapy, respectively (Table 2). Our meta-analysis revealed that the EGFR-TKIs plus anti-VEGFR combination increased PFS overall compared to EGFR-TKI alone (HR, 0.73; 95% CI: 0.61, 0.86; *p* = 0.0003, Figure 3). In the subgroup analyses, the EGFR-TKIs plus anti-VEGFR combination as a first line (HR, 0.64; 95% CI: 0.57, 0.71; *p* < 0.00001, (Figure 4A)) prolonged the PFS, whereas no improvement in PFS was observed with combination therapy as a second-line treatment (HR, 1.17; 95% CI, 0.79, 1.74; *p* = 0.44, (Figure 4B)) Significant heterogeneity was observed among studies in all analyses except for studies included in the case of the first line of treatment. 

Similarly, in another subgroup analysis, the first generation of the EGFR-TKIs plus anti-VEGFR combination did show significant improvement in PFS compared to monotherapy [HR, 0.63; 95% CI: 0.56, 0.71; *p* < 0.00001 (Figure 4C)]. Interestingly, the third generation of the EGFR-TKIs (osimertinib) plus anti-VEGFR combination did not show any improvement in PFS (HR, 1.09; 95% CI: 0.87, 1.36; *p* = 0.44) (Figure 4D) compared to EGFR-TKIs monotherapy. All analyses showed no significant heterogeneity among the studies included.

In addition, a significant improvement in PFS was observed when the EGFR-TKIs plus bevacizumab combination was compared with EGFR-TKIs alone (HR, 0.75; 95% CI: 0.61, 0.93; *p* = 0.01) (Supplementary Figure S1A). 

#### 3.4.2. Overall Survival

**Figure 5:**
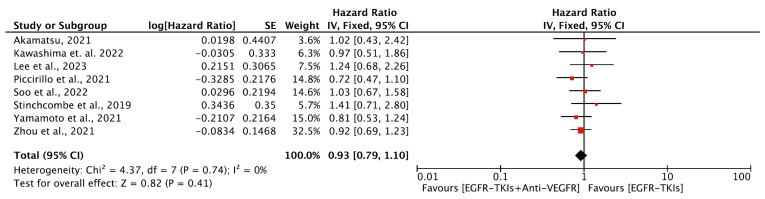
Meta-analysis showing the change in OS between EGFR-TKIs + Anti-VEGFR and EGFR-TKIs: fixed-effects model [19,20,23,24,27,29,35,38]. PFS: Progression Free survival; EGFR-TKIs = Epidermal Growth Factor Receptor Tyrosine Kinase Inhibitors; Anti-VEGFR: Anti-Vascular Endothelial Growth Factor Receptor; SE: Standard Error; df: degrees of freedom; CI: Confidence Interval.

**Figure 6:**
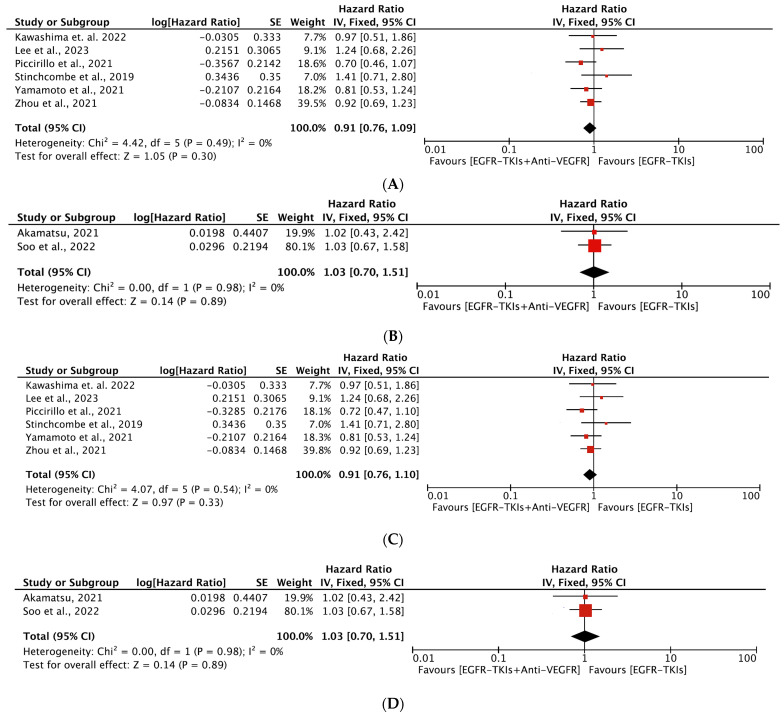
(**A**) Meta-analysis of subgroups showing the change in OS as a first line of treatment between EGFR-TKIs + Anti-VEGFR and EGFR-TKIs: fixed-effects model [19,20,23,24,29,35]. (**B**) Meta-analysis of subgroups showing the change in OS as a second line of treatment between EGFR-TKIs + Anti-VEGFR and EGFR-TKIs: fixed-effects model [27,38]. (**C**) Meta-analysis of subgroups showing the change in OS as a first generation (erlotinib and gefitinib) of EGFR-TKIs + Anti-VEGFR and EGFR-TKIs: fixed-effects model [19,20,23,24,29,35]. (**D**) Meta-analysis of subgroups showing the change in OS as a third generation (Osimertinib) of EGFR-TKIs + Anti-VEGFR and EGFR-TKIs: fixed-effects model [27,38]. OS: Overall survival; EGFR-TKIs= Epidermal Growth Factor Receptor Tyrosine Kinase Inhibitors; Anti-VEGFR: Anti-Vascular Endothelial Growth Factor Receptor; SE: Standard Error; df: degrees of freedom; CI: Confidence Interval.

A total of eight trials [19,20,23,24,27,29,35,38] included 1298 patients (649 each in combination and monotherapy group) investigating OS following the EGFR-TKIs plus anti-VEGFR combination compared to EGFR-TKI alone. Studies assessing OS as a first- and second-line treatment had 1062 and 236 patients, respectively. The median age of the patients in these studies ranged from 58 to 68 years. Five of the studies reporting OS were from Asia. Combination therapy was the first choice as a first-line agent in a majority (6) of these studies and had patient populations with L858R or del19 EGFR mutation. All included studies reported no statistically significant improvement in OS. The median OS ranged from 8.2 to 50.7 months for combination therapy and 7.6 to 50.6 months for monotherapy. The median OS for combination as first-line agent ranged from 32.4 to 50.7 months compared to 22.8 to 50.6 months for monotherapy. In comparison, the median OS was 8.2 to 24 months and 7.6 to 24.3 months as a second-line agent for the combination and monotherapy, respectively (Table 2). Our meta-analysis revealed that the EGFR-TKIs plus anti-VEGFR combination had no impact on OS compared with EGFR-TKI alone (HR, 0.93; 95% CI: 0.79, 1.10; *p* = 0.41, Figure 5). In the subgroup analyses, the EGFR-TKIs plus anti-VEGFR combination neither as a first-line (HR, 0.91; 95% CI: 0.76, 1.09; *p* = 0.30, Figure 6A) nor as a second-line treatment (HR, 1.03; 95% CI: 0.70, 1.51; *p* = 0.89, Figure 6B) prolonged the OS. No heterogeneity was observed among studies in all analyses.

In subgroup analyses, both generations of the EGFR-TKIs plus anti-VEGFR combination did not improve OS (HR, 0.91; 95% CI: 0.76, 1.10; *p* = 0.33 (Figure 6C); HR, 1.03; 95% CI: 0.70, 1.51; *p* = 0.89) (Figure 6D) over EGFR-TKI monotherapy. All analyses showed no significant heterogeneity among the studies included. The results were no different when the EGFR-TKIs plus bevacizumab combination was compared with EGFR-TKI alone (HR, 0.93; 95% CI: 0.79, 1.10; *p* = 0.41) (Supplementary Figure S1B).

#### 3.4.3. Objective Response Rate

**Figure 7:**
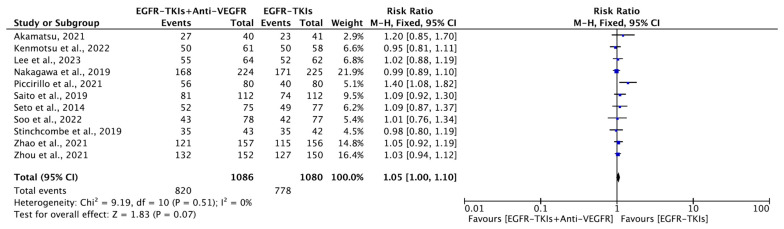
Meta-analysis showing the change in ORR between EGFR-TKIs + Anti-VEGFR and EGFR-TKIs: fixed-effects model [19,20,21,22,24,27,29,32,33,34,38]. PFS: Progression Free survival; EGFR-TKIs = Epidermal Growth Factor Receptor Tyrosine Kinase Inhibitors; Anti-VEGFR: Anti-Vascular Endothelial Growth Factor Receptor; SE: Standard Error; df: degrees of freedom; CI: Confidence Interval.

**Figure 8:**
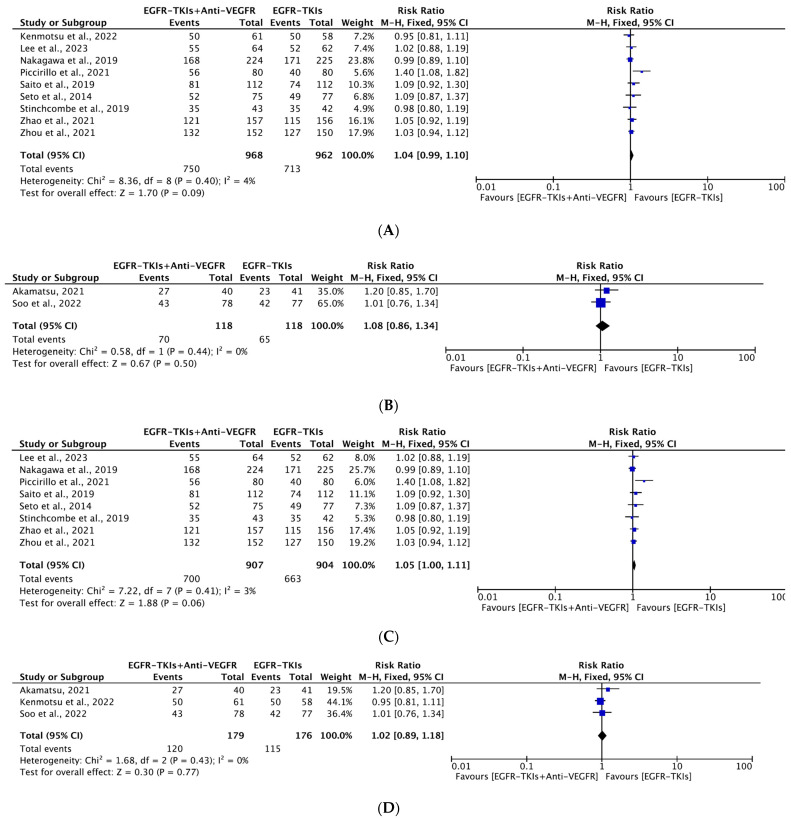
(**A**)**.** Meta-analysis of subgroups showing the change in ORR as a first line of treatment between EGFR-TKIs + Anti-VEGFR and EGFR-TKIs: fixed-effect model [19,20,21,22,24,29,32,33,34]. (**B**) Meta-analysis of subgroups showing the change in ORR as a second line of treatment between EGFR-TKIs + Anti-VEGFR and EGFR-TKIs: fixed-effects model [27,38]. (**C**) Meta-analysis of subgroups showing the change in ORR as a first generation (erlotinib and gefitinib) of EGFR-TKIs + Anti-VEGFR and EGFR-TKIs: fixed-effects model [19,20,21,22,24,29,32,34]. (**D**) Meta-analysis of subgroups showing the change in ORR as a third generation (Osimertinib) of EGFR-TKIs + Anti-VEGFR and EGFR-TKIs: fixed-effects model [27,33,38]. ORR: Objective Response Ratel; EGFR-TKIs = Epidermal Growth Factor Receptor Tyrosine Kinase Inhibitors; Anti-VEGFR: Anti-Vascular Endothelial Growth Factor Receptor; SE: Standard Error; df: degrees of freedom; CI: Confidence Interval.

All included studies reported ORR with 1086 participants in the EGFR-TKI plus anti-VEGFR combination group and 1080 participants in the EGFR-TKI monotherapy group. The majority (7) of them were conducted in Asia. The median age of patients ranged between 58 to 68 years. The majority (nine) of them had patient populations with L858R or del19 EGFR mutation, where combination therapy was mostly used as a first line of treatment. All studies reported no statistically significant improvement in ORR with the combination therapy. Interestingly, in a study by Piccirillo et al., EGFR-TKI monotherapy, in contrast to the EGFR-TKI plus anti-VEGFR combination, showed slightly improved ORR, although this improvement was not statistically significant. Our metanalysis revealed no significant improvement in ORR with combination therapy (RR, 1.05; 95% CI: 1.00, 1.10; *p* = 0.07; Figure 7). Similarly, in the subgroup analyses, the EGFR-TKI plus anti-VEGFR combination did not improve ORR both as a first line (RR, 1.04; 95% CI: 0.99, 1.10; *p* = 0.09) (Figure 8A) or as a second line (RR, 1.08; 95% CI: 0.86, 1.34; *p* = 0.50, Figure 8B) over EGFR-TKI monotherapy. The results of overall use and as a first and second-line treatment with combination showed no statistically significant improvement in ORR. All analyses showed no significant heterogeneity among the studies included. Similarly, in subgroup analyses, both generations of the EGFR-TKIs plus anti-VEGFR combination did not improve ORR (RR, 1.05; 95% CI: 1.00, 1.11; *p* = 0.06; RR, 1.02; 95% CI: 0.89, 1.18; *p* = 0.77) (Figure 8C,D) over EGFR-TKI monotherapy. All analyses showed no significant heterogeneity among the studies included.

A little improvement in ORR, although not statistically significant, was observed with EGFR-TKI alone compared to the EGFR-TKIs plus bevacizumab combination (RR, 1.07; 95% CI: 1.00, 1.14; *p* = 0.04) (Supplementary Figure S1C).

#### 3.4.4. Adverse Effects

The results of AEs related to the EGFR-TKIs plus anti-VEGFR combination group compared to the EGFR-TKI monotherapy group are presented in Table 3. The risk of grade 3 AEs and higher was greater in patients treated with the EGFR-TKIs plus anti-VEGFR combination (RR,3.27; 95% CI: 2.25, 4.75, *p* = <0.00001). Similarly, the risk of hypertension (RR, 5.11; 95% CI: 2.93, 8.89; *p* < 0.0001), diarrhea (RR, 2.25; 95% CI: 1.43, 3.54; *p* = 0.0005), and proteinuria (RR, 12.22; 95% CI: 5.83, 25.60; *p* < 0.0001) were significantly higher in NSCLC patients treated with EGFR-TKIs plus anti-VEGFR compared to EGFR-TKI alone (Table 3; Supplementary Figure S2A–E). However, skin rash (RR, 1.17; 95% CI: 0.99, 1.37; *p* = 0.06) was comparable between both groups. Significant heterogeneity was observed among the studies, except for studies reporting skin rash, diarrhea, and proteinuria.

Similar findings were obtained when the EGFR-TKIs plus bevacizumab combination was compared with EGFR-TKIs monotherapy. The risk of grade 3 AEs and higher hypertension and proteinuria were greater in patients treated with the EGFR-TKIs plus bevacizumab combination (Supplementary Tables S1 and S2; Supplementary Figure S3A–E). Significant heterogeneity was observed among the studies, except for studies reporting skin rash, proteinuria, and diarrhea.

### 3.5. Publication Bias

Assessment by funnel plot, Egger’s test, and Begg’s test (*p* > 0.05) showed no evidence of publication bias in our analyses.

### 3.6. Discussion

This meta-analysis evaluated 11 studies comprising 2182 patients with lung cancer and explored the efficacy and safety of EGFR-TKI plus anti-VEGFR agents as first and second-line treatment. In addition, we explored the improvement of survival outcomes across different generations of EGFR-TKIs versus EGFR-TKIs plus anti-VEGFR agent combinations and EGFR-TKIs in combination with bevacizumab only. Current clinical guidelines mandate the confirmation of EGFRm+ status before initiating therapy with EGFR-TKIs. Our results revealed that the combination of EGFR-TKI plus anti-VEGFR contributed to prolonging PFS compared to EGFR-TKI alone but did not impact the OS in advanced NSCLC treatment. Although several studies demonstrated significant benefits in PFS and ORR with new therapies, only a few showed favorable changes in OS.

PFS was improved with first-line EGFR-TKIs in combination with anti-VEGFR agents compared to EGFR-TKIs monotherapy, with 6 out of 9 studies demonstrating improved PFS in combination therapy [20,21,23,29,32,34]. However, there was no PFS improvement observed with EEGFR-TKIs combined with anti-VEGFR drugs as a second-line treatment; this was consistent with earlier clinical trials by Soo et al. and Akmatsu et al. [27,38]. PFS was improved with EGFR-TKIs in combination with bevacizumab compared to EGFR-TKIs monotherapy, particularly in the NEJ026 trial and several others [20,22,29,34], suggesting improved PFS with bevacizumab is potentially due to increasing drug distribution and tumor-suppressive effects. Combination therapy, particularly bevacizumab, showed significant PFS improvement in patients with concurrent TP53 mutations (CTONG1706 trial). Overall, PFS significantly increased with EGFR-TKIs plus anti-VEGFR compared to EGFR-TKI alone, particularly in first-line treatments, with median PFS ranging from 13.7 to 22.1 months. First-generation EGFR-TKIs combined with anti-VEGFR agents also improved PFS compared to monotherapy [20,21,23,29,32,34]. However, third-generation EGFR-TKIs (osimertinib) combined with anti-VEGFR agents did not enhance PFS compared to monotherapy, which is consistent with the results of previous trials [27,33,38].

Despite improvements in ORR and PFS, no OS benefits were observed across different lines of therapy and EGFR-TKI generations. Comorbidities and incomplete long-term follow-up data may have influenced the lack of OS benefits observed. Additionally, comprehensive OS data were lacking due to ongoing data collection and the nascent stage of OS analysis. Bevacizumab and erlotinib combination therapy did not substantially increase ORR compared to erlotinib monotherapy in two phase 3 trials (ARTEMIS and BEVERLY) [20,29]. This may have likely been influenced by post-study medication administration [20,29,36]. However, patients with verified EGFRm+ status experience notable PFS improvement with combination therapy, particularly in Asian populations, suggesting synergistic effects between bevacizumab and erlotinib in tumor suppression and delaying treatment resistance. However, further research is needed to comprehensively assess the clinical efficacy of these combinations. Additionally, the exclusion of patients with brain metastases in the BEVERLY study might have impacted the overall prognosis of the population [29]. While EGFR-TKIs and EGFR-TKI plus anti-VEGFR combination therapy are treatment options for advanced EGFRm+ NSCLC, most patients eventually acquire drug resistance and progress to relapse.

The incidence of AEs was higher in the EGFR-TKIs plus anti-VEGFR combination group than in the EGFR-TKIs alone group. Hypertension, skin rash, diarrhea, and proteinuria were the most common AEs believed to be dose-dependent [24,27,30,37,38]. Combination therapy often resulted in higher incidences of skin rash and proteinuria, which is likely due to VEGF-targeting medications. In the WJOG study, patients in the combination group had a greater incidence of proteinuria than those in the monotherapy group. A total of 55% of individuals receiving both drugs experienced grade 1–2 proteinuria, compared to 39% of those receiving osimertinib alone [33]. The small sample size and the previously documented history of Japanese patients being disproportionately impacted by proteinuria with anti-VEGF inhibitors restrict the validity of the data despite the high occurrence of this adverse event [38]. Diarrhea incidence in the combination group was also increased across multiple studies (CTONG1706, RELAY). There were reports of 73.2% diarrhea incidence of varying grades in the combination group compared to 51.9% in the monotherapy group [21,32]. In addition to the previously described AEs, combination therapy occasionally caused toxicity-related AEs that resulted in patients discontinuing the medication, with bevacizumab discontinuation being more frequent in longer treatment durations. Nonetheless, most of the toxicities associated with this combination therapy were considered tolerable and manageable. Patients who received ramucirumab with erlotinib similarly showed a longer PFS than the monotherapy group, suggesting initial targeting of VEGFR2 and EGFR pathways is a viable option for the NSCLC treatment [33]. Interim OS analysis indicated no AEs with ramucirumab plus erlotinib, aligning with current guidelines recommending erlotinib in combination with VEGFR-targeted monoclonal antibodies as first-line therapy for advanced NSCLC.

The present study is not without limitations. Variations in study designs, agents used, and patient demographics, including gender and race/ethnicity, may have impacted survival outcomes. The exclusion of studies with incomplete survival outcomes data and lack of consideration for second and third-generation EGFR-TKIs in combination with other targeted agents limited inclusion in the meta-analysis. Additionally, clinical trials involving second and third-generation EGFR-TKIs with other targeted agents were not considered. Subgroup analysis was exploratory, requiring confirmation from future randomized controlled trials (RCTs). Nevertheless, the meta-analysis included high-quality RCTs without indication of publication bias, providing compelling evidence for the safety and efficacy of EGFR-TKIs plus anti-VEGFR combination therapy in advanced NSCLC patients.

## 4. Conclusions

The results of this meta-analysis showed that a combination of EGFR-TKIs plus anti-VEGFR- agents prolonged PFS and ORR in patients with advanced NSCLC but failed to significantly improve OS. This combination strategy also resulted in common ADEs such as hypertension, skin rash, diarrhea, and proteinuria. EGFR-TKIs plus anti-VEGFR combination can be recommended as a therapeutic strategy for patients with advanced NSCLC.

## Figures and Tables

**Table 1:** Characteristics of the included studies.

Author, Year	EGFR-TKI	Anti-VEGFR Agent	Phase	Study Region	Trial Name	EGFR Mutation	Line of Treatment	Prior Treatment	Patients (N)	Age (yrs.) (Median)	Female, (%)	% Asian	ITT Analysis
Akamatsu, 2021 [38]	Osimertinib	Bevacizumab	II	Asia	WJOG8715L	T790M EGFR	Second-line	EGFR-TKI	81	68	59	100	+
^a^ Kawashima et. al., 2022 [35] ^a^ Saito et al., 2019 [34]	Erlotinib	Bevacizumab	III	Asia	NEJ026	L858R or del19 EGFR	First-line	None	224	67	64	100	+
Kenmotsu et al., 2022 [33]	Osimertinib	Bevacizumab	II	Asia	WJOG9717L	L858R or del19 EGFR	First-line	None	122	67	61	100	+
Nakagawa et al., 2019 [32]	Erlotinib	Ramucirumab	III	Global	RELAY	L858R or del19 EGFR	First-line	None	449	64	63	77	+
Piccirillo et al., 2022 [29]	Erlotinib	Bevacizumab	III	Europe	BEVERLY	L858R or del19 EGFR	First-line	None	160	67	64	0	+
Soo et al., 2022 [27]	Osimertinib	Bevacizumab	II	Global	BOOSTER	T790M EGFR	Second line	EGFR-TKI	155	67	62	41	+
Stinchcombe et al., 2019 [24]	Erlotinib	Bevacizumab	II	US	NCT01532089	L858R or del19 EGFR	First-line	None	88	63	70	3	+
^b^ Yamamoto et al., 2021 [23] ^b^ Seto et al. 2014 [22]	Erlotinib	Bevacizumab	II	Asia	JO25567	L858R or del19 EGFR	First-line	None	152	67	63	100	
Zhao et al., 2021 [21]	Gefitinib	Apatinib	III	Asia	ACTIVE CTONG 1706	L858R or del19 EGFR	First line	None	313	59	59	100	+
Zhou et al., 2021 [20]	Erlotinib	Bevacizumab	III	Asia	ARTEMIS CTOG1509	L858R or del19 EGFR	First-line	None	311	58	62	100	+
Lee et al., 2023 [19]	Erlotinib	Bevacizumab	II	Asia	NCT03126799	L858R or del19 EGFR	First-line	None	127	63	66	100	+

^a^ Dual publication of NEJ026 trial; ^b^ dual publication of JO25567 trial; Global = US/Canada/Other; Asia = Japan/China; Europe (Italy); EGFR-TKI = Epidermal Growth Factor Receptor Tyrosine Kinase Inhibitor; Anti-VEGFR = Anti-Vascular Endothelial Growth Factor Receptor; ITT = Intention to Treat Analysis; EGFR = Epidermal Growth Factor Receptor; + = Yes.

**Table 2:** Survival of patients receiving EGFR-TKIs plus anti-VEGFR- combination and EGFR-TKI alone.

Author, Year	ORR—Comb	Patient— Comb (N)	ORR—Mono	Patients— Mono (N)	ORR—*p*-Value	Comb—PFS (Months)	Mono—PFS (Months)	PFS—HR, (95% CI)	Comb—OS (Months)	Mono—OS (Months)	OS—HR, (95% CI)
Akamatsu, 2021 [38]	27	40	23	41	0.2	9.4	13.5	1.44 [1.00–2.08]	8.2	7.6	1.02 [0.43, 2.44]
^a^ Kawashima et. al., 2022 [35]									50.7	46.2	0.970 [0.505–1.866]
^a^ Saito et al., 2019 [34]	81	112	74	112	0.31	16.9	13.3	0.61 [0.42–0.88]			
Kenmotsu et al., 2022 [33]	50	61	50	58	0.786	22.1	20.2	0.862 [0.531–1.397]			
Nakagawa et al., 2019 [32]	168	224	171	225		19.4	12.4	0.59 [0.46–0.76]			
Piccirillo et al., 2022 [29]	56	80	40	80	0.01	15.4	9.6	0.66 [0.47–0.92]	33.3	22.8	0.72 [0.47–1.10]
Soo et al., 2022 [27]	43	78	42	77	0.93	15.4	12.3	0.96 [0.68–1.37]	24	24.3	1.03 (0.67–1.56)
Stinchcombe et al., 2019 [24]	35	43	35	42	0.81	17.9	13.5	0.81 [0.50–1.31]	32.4	50.6	1.41 [0.71–2.81]
^b^ Yamamoto et al., 2021 [23]						16.4	9.8	0.52 (0.35–0.76)	47	47.4	0.81 [0.53–1.23]
^b^ Seto et al., 2014 [22]	52	75	49	77	0.49						
Zhao et al., 2021 [21]	121	157	115	156	0.56	13.7	10.2	0.71 [0.54–0.95]			
Zhou et al., 2021 [20]	132	152	127	150	0.56	17.9	11.2	0.55 [0.41–0.73]	36.2	31.6	0.92 [0.69, 1.23]
Lee et al., 2023 [19]	55	64	52	62	0.48	17.5	12.4	0.74 [0.51, 1.08]			1.24 [0.68, 2.26]

^a^ Dual publication of NEJ026 trial; ^b^ dual publication of JO25567 trial; EGFR-TKIs = Epidermal Growth Factor Receptor Tyrosine Kinase Inhibitors; ORR = Objective Response Rate (Complete Response (CR) + Partial Response (PR); Mono = Monotherapy; Comb = Combination Therapy; PFS = Progression Free Survival; HR = Hazard Ratio; CI = Confidence Interval; OS = Overall Survival.

**Table 3:** Relative risk of adverse drug events in patients with advanced NSCLC treated with EGFR-TKIs plus Anti-VEGFR combination compared to EGFR-TKIs alone.

Adverse Drug Events	EGFR-TKIs + Anti-VEGFR Event/Total	EGFR-TKIs Event/Total	RR (95% CI)	*p*-Value	Heterogeneity	
					I^2^	*p*-Value
Grade 3 AEs	537/1086 (49.4%)	154/1088 (14.2%)	3.27 (2.25, 4.75)	<0.00001	82%	<0.00001
Skin rash	154/1.010 (15.2%)	132/1011 (13.1%)	1.17 (0.99, 1.37)	0.06	39%	0.10
Hypertension	282/1.086 (25.9%)	47/1088 (4.3%)	5.11 (2.93, 8.89)	<0.00001	62%	0.003
Diarrhea	57/1086 (5.2%)	25/1088 (2.3%)	2.25 (1.43, 3.54)	0.0005	25%	0.20
Proteinuria	88/1086 (8.1%)	4/1088 (0.4%)	12.22 (5.83, 25.60)	<0.00001	0%	0.96

NSCLC: Non-Small Cell Lung Cancer; EGFR-TKIs = Epidermal Growth Factor Receptor Tyrosine Kinase Inhibitors; AEs: Adverse Events; Anti-VEGFR: Anti-Vascular Endothelial Growth Factor Receptor; RR: Relative Risk; CI: Confidence Interval.

## Data Availability

All available data can be accessed by contacting the corresponding author.

## Supplementary Materials

The following supporting information can be downloaded at: https://www.mdpi.com/article/10.3390/cancers16061188/s1, File S1: Figure S1: (A) Meta-analysis of PFS and change in PFS between EGFR-TKIs plus Bevacizumab and EGFR-TKIs: random-effects model [19,20,23,24,27,29,33,34,38] (B) Meta-analysis of OS and change in OS between EGFR-TKIs plus Bevacizumab and EGFR-TKIs: fixed-effects model [19,20,23,24,27,29,35,38]. (C) Meta-analysis of ORR and change in ORR between EGFR-TKIs plus Bevacizumab and EGFR-TKIs: fixed-effects model [19,20,22,24,27,29,33,34,38]; Figure S2: (A) Risk ratio of grade 3 and higher AEs between EGFR-TKIs + Anti-VEGFR and EGFR-TKIs: random-effects model [19,20,21,22,24,27,29,32,33,34,38]; (B) Risk ratio of hypertension between EGFR-TKIs + Anti-VEGFR and EGFR-TKIs: random-effects model [19,20,21,22,24,27,29,32,33,34,38]; (C) Risk ratio of skin rash between EGFR-TKIs + Anti-VEGFR and EGFR-TKIs: fixed-effects model [19,20,21,22,24,29,32,33,34,38]; (D) Risk ratio of proteinuria between EGFR-TKIs + Anti-VEGFR and EGFR-TKIs: fixed-effects model [19,20,21,22,24,27,29,32,33,34,38]; (E) Risk ratio of diarrhea between EGFR-TKIs + Anti-VEGFR and EGFR-TKIs: fixed-effects model [19,20,21,22,24,27,29,32,33,34,38]; Figure S3: (A) Risk ratio of grade 3 and higher AEs between EGFR-TKIs plus Bevacizumab and EGFR-TKIs: random-effects model [19,20,22,24,27,29,33,34,38]; (B) Risk ratio of hypertension between EGFR-TKIs plus Bevacizumab and EGFR-TKIs: random-effects model [19,20,22,24,27,29,33,34,38]; (C) Risk ratio of skin rash between EGFR-TKIs plus Bevacizumab and EGFR-TKIs: fixed-effects model [19,20,22,24,29,33,34,38]; (D) Risk ratio of proteinuria between EGFR-TKIs plus Bevacizumab and EGFR-TKIs: fixed-effects model [19,20,22,24,27,29,33,34,38]; (E) Risk ratio of diarrhea between EGFR-TKIs plus Bevacizumab and EGFR-TKIs: fixed-effects model [19,20,22,24,27,29,33,34,38]; Table S1: Adverse events in patients receiving EGFR-TKIs plus anti VEGFR- TKI combination and EGFR-TKI alone [19,20,21,22,24,27,29,32,33,34,38]; Table S2: Relative risk of adverse events in patients with advanced NSCLC treated with EGFR-TKIs plus Bevacizumab combination compared to EGFR-TKIs alone; Table S3: Demographic characteristics of the patients in studies that were a part of systematic review (qualitative synthesis) in additions to ones that were included in the meta-analyses [25,26,28,30,31,36,37]; File S2: (A) PRISMA 2020 Checklist [19,20,21,22,23,24,25,26,27,28,29,30,31,32,33,34,35,36,37,38,39]; (B) PubMed Search Strategy; (C) Content of Data Abstraction Form.
