# Supplementary material for: Investigating the Efficacy of EGFR-TKIs and Anti-VEGFR Combination in Advanced Non-Small Cell Lung Cancer: A Meta-Analysis
Source: Cancers (Basel). 2024 Mar 18;16(6):1188. doi: 10.3390/cancers16061188 (PMC10969009; doi:10.3390/cancers16061188)
Supplement: Supplementary file 1 [file cancers-16-01188-s001.zip › cancers-2889423-supplementary/cancers-2889423_Proof Supplimentary File S2.pdf]

File S2 (A): PRISMA 2020 Checklist.

Section and Topic	Item #	Checklist item	Location where item is reported
**TITLE**			
Title	1	Identify the report as a systematic review/Meta-analysis	Page 1
**ABSTRACT**			
Abstract	2	See the PRISMA 2020 for Abstracts checklist.	Page 2
**INTRODUCTION**			
Rationale	3	Describe the rationale for the review in the context of existing knowledge.	Page 4-6
Objectives	4	Provide an explicit statement of the objective(s) or question(s) the review addresses.	Page 6
**METHODS**			
Eligibility criteria	5	Specify the inclusion and exclusion criteria for the review and how studies were grouped for the syntheses.	Page 6-7
Information sources	6	Specify all databases, registers, websites, organisations, reference lists and other sources searched or consulted to identify studies. Specify the date when each source was last searched or consulted.	Page 6-8
Search strategy	7	Present the full search strategies for all databases, registers and websites, including any filters and limits used.	Page 6-7; Supplementary figure S1B and S1C
Selection process	8	Specify the methods used to decide whether a study met the inclusion criteria of the review, including how many reviewers screened each record and each report retrieved, whether they worked independently, and if applicable, details of automation tools used in the process.	Page 6-8, Figure 1
Data collection process	9	Specify the methods used to collect data from reports, including how many reviewers collected data from each report, whether they worked independently, any processes for obtaining or confirming data from study investigators, and if applicable, details of automation tools used in the process.	Page 6-7; Supplementary figure S1B and C
Data items	10a	List and define all outcomes for which data were sought. Specify whether all results that were compatible with each outcome domain in each study were sought (e.g. for all measures, time points, analyses), and if not, the methods used to decide which results to collect.	Page 6-8, Supplementary figure S1B and S1C
	10b	List and define all other variables for which data were sought (e.g. participant and intervention characteristics, funding sources). Describe any assumptions made about any missing or unclear information.	Page 6-7, Supplementary figure

Section and Topic	Item #	Checklist item	Location where item is reported
			S1B and S1C
Study risk of bias assessment	11	Specify the methods used to assess risk of bias in the included studies, including details of the tool(s) used, how many reviewers assessed each study and whether they worked independently, and if applicable, details of automation tools used in the process.	Page 6-7, Fig. 2A & B
Effect measures	12	Specify for each outcome the effect measure(s) (e.g. risk ratio, mean difference) used in the synthesis or presentation of results.	Page 6-7
Synthesis methods	13a	Describe the processes used to decide which studies were eligible for each synthesis (e.g. tabulating the study intervention characteristics and comparing against the planned groups for each synthesis (item #5)).	Page 6-7
	13b	Describe any methods required to prepare the data for presentation or synthesis, such as handling of missing summary statistics, or data conversions.	Page 6-7
	13c	Describe any methods used to tabulate or visually display results of individual studies and syntheses.	Page 7
	13d	Describe any methods used to synthesize results and provide a rationale for the choice(s). If meta-analysis was performed, describe the model(s), method(s) to identify the presence and extent of statistical heterogeneity, and software package(s) used.	Page 7
	13e	Describe any methods used to explore possible causes of heterogeneity among study results (e.g. subgroup analysis, meta-regression).	Page 7
	13f	Describe any sensitivity analyses conducted to assess robustness of the synthesized results.	Page 7
Reporting bias assessment	14	Describe any methods used to assess risk of bias due to missing results in a synthesis (arising from reporting biases).	Page 6
Certainty assessment	15	Describe any methods used to assess certainty (or confidence) in the body of evidence for an outcome.	Page 7
**RESULTS**			
Study selection	16a	Describe the results of the search and selection process, from the number of records identified in the search to the number of studies included in the review, ideally using a flow diagram.	Page 9, Figure 1
	16b	Cite studies that might appear to meet the inclusion criteria, but which were excluded, and explain why they were excluded.	Page 9, Figure 1
Study	17	Cite each included study and present its characteristics.	Page 9-10, Fig. 2A and

Section and Topic	Item #	Checklist item	Location where item is reported
characteristics			B, Table 1 - 4
Risk of bias in studies	18	Present assessments of risk of bias for each included study.	Page 9-10, Fig. 2A and B
Results of individual studies	19	For all outcomes, present, for each study: (a) summary statistics for each group (where appropriate) and (b) an effect estimate and its precision (e.g. confidence/credible interval), ideally using structured tables or plots.	Table 2 and 4, Fig 3 – 5, supplementary Figure S1A-S1E
Results of syntheses	20a	For each synthesis, briefly summarise the characteristics and risk of bias among contributing studies.	Page 9-11
	20b	Present results of all statistical syntheses conducted. If meta-analysis was done, present for each the summary estimate and its precision (e.g. confidence/credible interval) and measures of statistical heterogeneity. If comparing groups, describe the direction of the effect.	Page 5-10, Fig.3 – 5, Supplementary Fig S1A – S1E
	20c	Present results of all investigations of possible causes of heterogeneity among study results.	Page 9-13
	20d	Present results of all sensitivity analyses conducted to assess the robustness of the synthesized results.	Page 9-13
Reporting biases	21	Present assessments of risk of bias due to missing results (arising from reporting biases) for each synthesis assessed.	Page 9--13
Certainty of evidence	22	Present assessments of certainty (or confidence) in the body of evidence for each outcome assessed.	Page 9-13
**DISCUSSION**			
Discussion	23a	Provide a general interpretation of the results in the context of other evidence.	Page 14-20
	23b	Discuss any limitations of the evidence included in the review.	Page 14-20
	23c	Discuss any limitations of the review processes used.	Page 14-20
	23d	Discuss implications of the results for practice, policy, and future research.	Page 14-20
**OTHER INFORMATION**			
Registration and protocol	24a	Provide registration information for the review, including register name and registration number, or state that the review was not registered.	OSF, Page 6
	24b	Indicate where the review protocol can be accessed, or state that a protocol was not prepared.	Page 6
	24c	Describe and explain any amendments to information provided at registration or in the protocol.	NA
Support	25	Describe sources of financial or non-financial support for the review, and the role of the funders or sponsors in the	Page 20

Section and Topic	Item #	Checklist item	Location where item is reported
		review.	
Competing interests	26	Declare any competing interests of review authors.	Page 20
Availability of data, code and other materials	27	Report which of the following are publicly available and where they can be found: template data collection forms; data extracted from included studies; data used for all analyses; analytic code; any other materials used in the review.	Page 20

From: Page MJ, McKenzie JE, Bossuyt PM, Boutron I, Hoffmann TC, Mulrow CD, et al. The PRISMA 2020 statement: an updated guideline for reporting systematic reviews. BMJ 2021;372:n71. doi: 10.1136/bmj.n71
For more information, visit: http://www.prisma-statement.org/

File S2B: PubMed Search Strategy.

(((("Epidermal Growth Factor Receptor Protein-Tyrosine Kinase"[Title/Abstract] OR "Epidermal Growth Factor Receptor Protein-Tyrosine Kinase"[Title/Abstract] OR "epidermal growth factor"[Title/Abstract] OR "epidermal growth factor receptor"[Title/Abstract] OR "receptor egf"[Title/Abstract] OR "egf receptor*"[Title/Abstract] OR "receptor epidermal growth factor"[Title/Abstract] OR "EGR"[Title/Abstract] OR "EGFR"[Title/Abstract] OR "ErbB-1"[Title/Abstract] OR "HER1"[Title/Abstract] OR "egfr tki"[Title/Abstract] OR "Gefitinib"[Title/Abstract] OR "Erlotinib"[Title/Abstract] OR "Afatinib"[Title/Abstract] OR "Osimertinib"[Title/Abstract] OR "egfr tyrosine kinase inhibitor*"[Title/Abstract]) AND "Angiogenesis Inhibitors"[MeSH Terms]) OR (("Angiogenesis Inhibitors"[Title/Abstract] OR ("angiogen*"[All Fields] AND "Antagonists"[Title/Abstract]) OR ("angiogen*"[All Fields] AND "Inhibitors"[Title/Abstract]) OR (("antagonists and inhibitors"[MeSH Subheading] OR ("Antagonists"[All Fields] AND "Inhibitors"[All Fields]) OR "antagonists and inhibitors"[All Fields] OR "Inhibitors"[All Fields] OR "inhibitor"[All Fields] OR "inhibitor s"[All Fields]) AND "angiogenesis factor"[Title/Abstract]) OR (("antagonists and inhibitors"[MeSH Subheading] OR ("Antagonists"[All Fields] AND "Inhibitors"[All Fields]) OR "antagonists and inhibitors"[All Fields] OR "Inhibitors"[All Fields] OR "inhibitor"[All Fields] OR "inhibitor s"[All Fields]) AND "Neovascularization"[Title/Abstract]) OR (("Angiogenesis"[Journal] OR "Angiogenesis"[All Fields]) AND "factor inhibitors"[Title/Abstract]) OR (("Factor"[All Fields] OR "factor s"[All Fields] OR "factors"[All Fields]) AND "inhibitors angiogenesis"[Title/Abstract]) OR "bevacizumab"[Title/Abstract] OR "Anlotinib"[Title/Abstract] OR "Ramucirumab"[Title/Abstract] OR "endostatin*"[Title/Abstract]) AND ("carcinoma, non small cell lung"[MeSH Terms] OR ("carcinoma non small cell lung"[Title/Abstract] OR "carcinoma non small cell lung"[Title/Abstract] OR "lung carcinoma non small cell"[Title/Abstract] OR "nonsmall cell lung cancer"[Title/Abstract] OR "non small cell lung carcinoma*"[Title/Abstract] OR "non small cell lung carcinoma"[Title/Abstract] OR "carcinoma non small cell lung"[Title/Abstract] OR "non small cell lung cancer"[Title/Abstract] OR "NSCLC"[Title/Abstract])))) AND ("clinical study"[Publication Type] OR "clinical trial"[Publication Type])) AND ((clinicalstudy[Filter] OR clinicaltrial[Filter] OR controlledclinicaltrial[Filter] OR randomizedcontrolledtrial[Filter]) AND (humans[Filter]) AND (english[Filter]))

File S2C: Content of Data Abstraction Form

1. Citation
2. Trial name/number
3. First author
4. Year of publication
5. Country of origin
6. Study type
7. Selection of Participant
8. Concealment
9. Blinding
10. Sample size
11. Ethnicity
12. Number of patients in each group
13. Staging of cancer
14. Line of treatment
15. Type of EFGR-TKIs Agent
16. Type of Anti-VEFGR Agent
17. Type of EGFR mutation
18. Prior treatment
19. Type of analysis (ITT/mITT)
20. Intervention
21. Comparison
22. Survival outcomes (PFS, OS, ORR with 95%CI and p-values)
23. Number of adverse events in each group for grade 3 and higher adverse events, skin rash, hypertension, proteinuria, and diarrhea.
24. Study limitations
25. Disclosures/Funding
26. Comments/Remarks if any
