# Supplementary material for: Investigating the Efficacy of EGFR-TKIs and Anti-VEGFR Combination in Advanced Non-Small Cell Lung Cancer: A Meta-Analysis
Source: Cancers (Basel). 2024 Mar 18;16(6):1188. doi: 10.3390/cancers16061188 (PMC10969009; doi:10.3390/cancers16061188)
Supplement: Supplementary file 1 [file cancers-16-01188-s001.zip › cancers-2889423-supplementary/cancers-2889423_Proof_Supplimentary File S1.pdf]

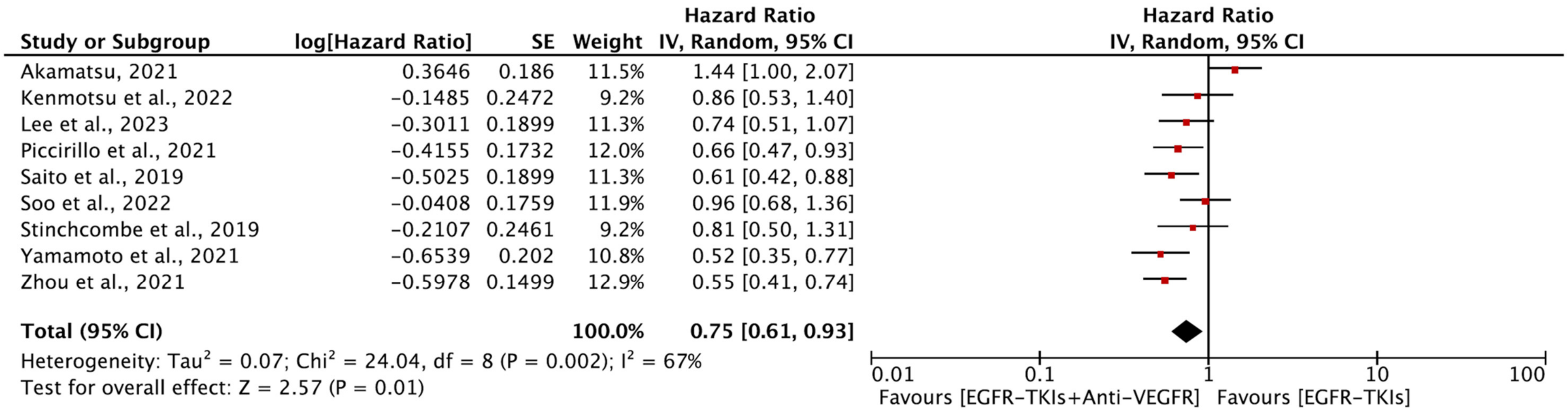
Study or Subgroup
log[Hazard Ratio]
SE
Weight
Hazard Ratio IV, Random, 95% CI
Hazard Ratio IV, Random, 95% CI
Akamatsu, 2021 0.3646 0.186 11.5% 1.44 [1.00, 2.07]
Kenmotsu et al., 2022 −0.1485 0.2472 9.2% 0.86 [0.53, 1.40]
Lee et al., 2023 −0.3011 0.1899 11.3% 0.74 [0.51, 1.07]
Piccirillo et al., 2021 −0.4155 0.1732 12.0% 0.66 [0.47, 0.93]
Saito et al., 2019 −0.5025 0.1899 11.3% 0.61 [0.42, 0.88]
Soo et al., 2022 −0.0408 0.1759 11.9% 0.96 [0.68, 1.36]
Stinchcombe et al., 2019 −0.2107 0.2461 9.2% 0.81 [0.50, 1.31]
Yamamoto et al., 2021 −0.6539 0.202 10.8% 0.52 [0.35, 0.77]
Zhou et al., 2021 −0.5978 0.1499 12.9% 0.55 [0.41, 0.74]
Total (95% CI) 100.0% 0.75 [0.61, 0.93]
Heterogeneity: Tau² = 0.07; Chi² = 24.04, df = 8 (P = 0.002); I² = 67%
Test for overall effect: Z = 2.57 (P = 0.01)
0.01
0.1
1
10
100
Favours [EGFR−TKIs+Anti−VEGFR]
Favours [EGFR−TKIs]

(**A**)

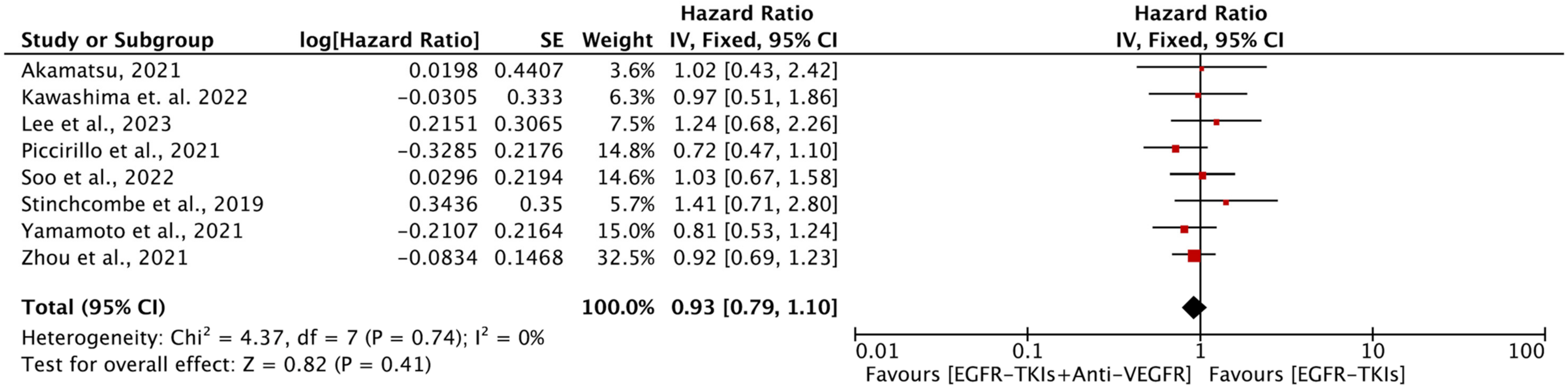
Study or Subgroup
log[Hazard Ratio]
SE
Weight
Hazard Ratio IV, Fixed, 95% CI
Hazard Ratio IV, Fixed, 95% CI
Akamatsu, 2021 0.0198 0.4407 3.6% 1.02 [0.43, 2.42]
Kawashima et. al. 2022 −0.0305 0.333 6.3% 0.97 [0.51, 1.86]
Lee et al., 2023 0.2151 0.3065 7.5% 1.24 [0.68, 2.26]
Piccirillo et al., 2021 −0.3285 0.2176 14.8% 0.72 [0.47, 1.10]
Soo et al., 2022 0.0296 0.2194 14.6% 1.03 [0.67, 1.58]
Stinchcombe et al., 2019 0.3436 0.35 5.7% 1.41 [0.71, 2.80]
Yamamoto et al., 2021 −0.2107 0.2164 15.0% 0.81 [0.53, 1.24]
Zhou et al., 2021 −0.0834 0.1468 32.5% 0.92 [0.69, 1.23]
Total (95% CI) 100.0% 0.93 [0.79, 1.10]
Heterogeneity: Chi² = 4.37, df = 7 (P = 0.74); I² = 0%
Test for overall effect: Z = 0.82 (P = 0.41)
0.01
0.1
1
10
100
Favours [EGFR−TKIs+Anti−VEGFR]
Favours [EGFR−TKIs]

(**B**)

Study or Subgroup	EGFR−TKIs+Anti−VEGFR Events	Total	EGFR−TKIs Events	Total	Weight	Risk Ratio M−H, Fixed, 95% CI
Akamatsu, 2021	27	40	23	41	4.6%	1.20 [0.85, 1.70]
Kenmotsu et al., 2022	50	61	50	58	10.4%	0.95 [0.81, 1.11]
Lee et al., 2023	55	64	52	62	10.7%	1.02 [0.88, 1.19]
Piccirillo et al., 2021	56	80	40	80	8.1%	1.40 [1.08, 1.82]
Saito et al., 2019	81	112	74	112	15.0%	1.09 [0.92, 1.30]
Seto et al., 2014	52	75	49	77	9.8%	1.09 [0.87, 1.37]
Soo et al., 2022	43	78	42	77	8.5%	1.01 [0.76, 1.34]
Stinchcombe et al., 2019	35	43	35	42	7.2%	0.98 [0.80, 1.19]
Zhou et al., 2021	132	152	127	150	25.8%	1.03 [0.94, 1.12]
**Total (95% CI)**		**705**		**699**	**100.0%**	**1.07 [1.00, 1.14]**
Total events	531		492			

Heterogeneity: $Chi^2 = 8.77$, df = 8 (P = 0.36); $I^2 = 9\%$
Test for overall effect: Z = 2.08 (P = 0.04)

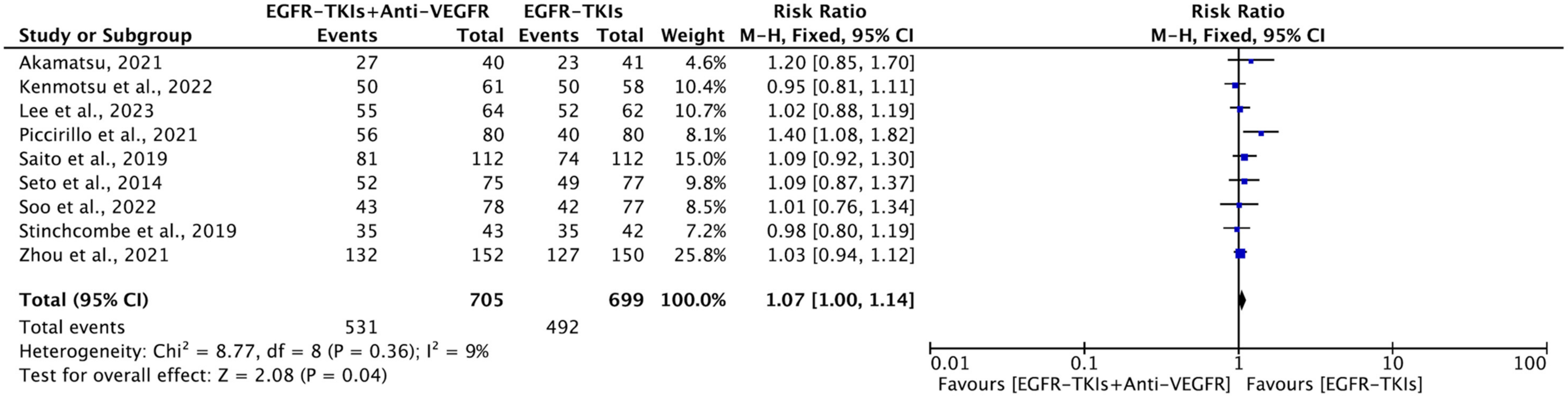

(**C**)

Supplimentary Figure S1: (A): Meta-analysis of PFS and change in PFS between EFGR-TKIs plus Bevacizumab and EGFR-TKIs: random-effects model [19,20,23,24,27,29,33,34,38]. (B): Meta-analysis of OS and change in OS between EFGR-TKIs plus Bevacizumab and EGFR-TKIs: fixed-effects model [19,20,23,24,27,29,35,38]. (C): Meta-analysis of ORR and change in ORR between EFGR-TKIs plus Bevacizumab and EGFR-TKIs: fixed-effects model [19,20,22,24,27,29,33,34,38].

Study or Subgroup	EGFR-TKIs+Anti-VEGFR Events	EGFR-TKIs+Anti-VEGFR Total	EGFR-TKIs Events	EGFR-TKIs Total	Weight	Risk Ratio M-H, Random, 95% CI
Akamatsu, 2021	19	40	3	41	5.8%	6.49 [2.08, 20.24]
Kenmotsu et al., 2022	6	61	5	60	5.9%	1.18 [0.38, 3.66]
Lee et al., 2023	29	64	6	63	8.0%	4.76 [2.12, 10.67]
Nakagawa et al., 2019	76	221	20	225	10.7%	3.87 [2.45, 6.11]
Piccirillo et al., 2021	55	80	21	79	11.1%	2.59 [1.74, 3.84]
Saito et al., 2019	63	112	28	114	11.3%	2.29 [1.60, 3.29]
Seto et al., 2014	71	75	24	77	11.5%	3.04 [2.17, 4.25]
Soo et al., 2022	6	76	4	77	5.4%	1.52 [0.45, 5.17]
Stinchcombe et al., 2019	37	43	23	45	11.6%	1.68 [1.23, 2.30]
Zhao et al., 2021	123	157	10	154	9.5%	12.06 [6.59, 22.09]
Zhou et al., 2021	52	157	10	153	9.2%	5.07 [2.67, 9.60]
**Total (95% CI)**		**1086**		**1088**	**100.0%**	**3.27 [2.25, 4.75]**
Total events	537		154			

Heterogeneity: $Tau^2 = 0.29$; $Chi^2 = 54.48$, df = 10 (P < 0.00001); $I^2 = 82\%$
Test for overall effect: Z = 6.20 (P < 0.00001)

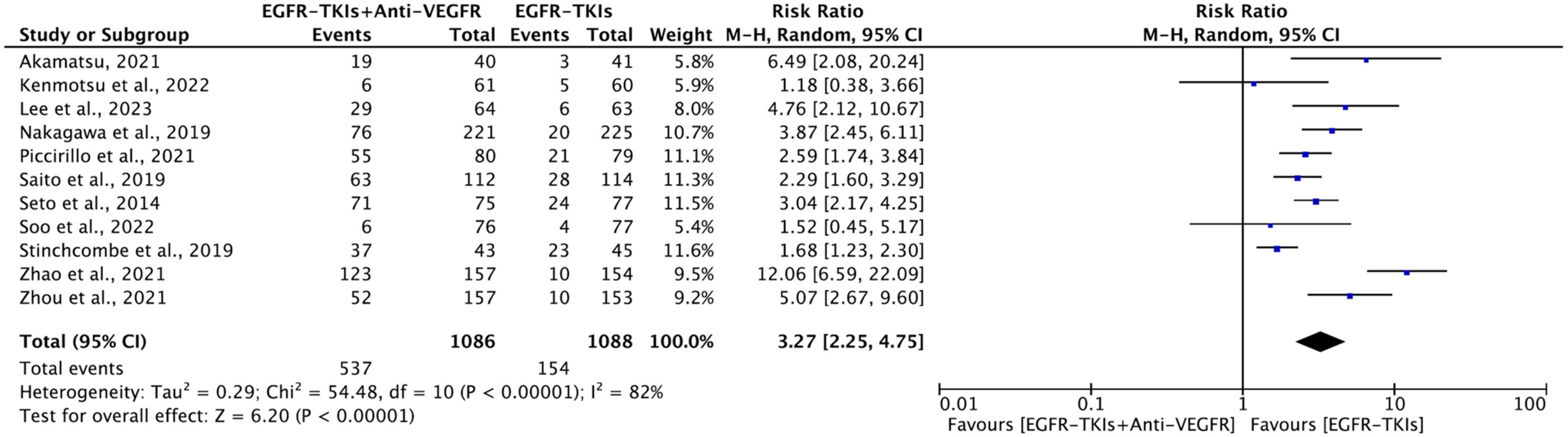

(**A**)

Study or Subgroup	EGFR-TKIs+Anti-VEGFR Events	EGFR-TKIs+Anti-VEGFR Total	EGFR-TKIs Events	EGFR-TKIs Total	Weight	Risk Ratio M-H, Random, 95% CI
Akamatsu, 2021	8	40	0	41	3.2%	17.41 [1.04, 292.02]
Kenmotsu et al., 2022	4	61	3	60	8.0%	1.31 [0.31, 5.61]
Lee et al., 2023	9	64	0	63	3.2%	18.71 [1.11, 314.73]
Nakagawa et al., 2019	52	221	12	225	14.8%	4.41 [2.42, 8.04]
Piccirillo et al., 2021	19	80	4	79	11.1%	4.69 [1.67, 13.17]
Saito et al., 2019	26	112	1	114	5.5%	26.46 [3.65, 191.71]
Seto et al., 2014	45	75	8	77	14.1%	5.78 [2.92, 11.42]
Soo et al., 2022	0	76	1	77	2.6%	0.34 [0.01, 8.16]
Stinchcombe et al., 2019	17	43	9	45	14.0%	1.98 [0.99, 3.95]
Zhao et al., 2021	73	157	4	154	11.5%	17.90 [6.71, 47.77]
Zhou et al., 2021	29	157	5	153	12.0%	5.65 [2.25, 14.22]
**Total (95% CI)**		**1086**		**1088**	**100.0%**	**5.11 [2.93, 8.89]**
Total events	282		47			

Heterogeneity: $Tau^2 = 0.45$; $Chi^2 = 26.21$, df = 10 (P = 0.003); $I^2 = 62\%$
Test for overall effect: Z = 5.76 (P < 0.00001)

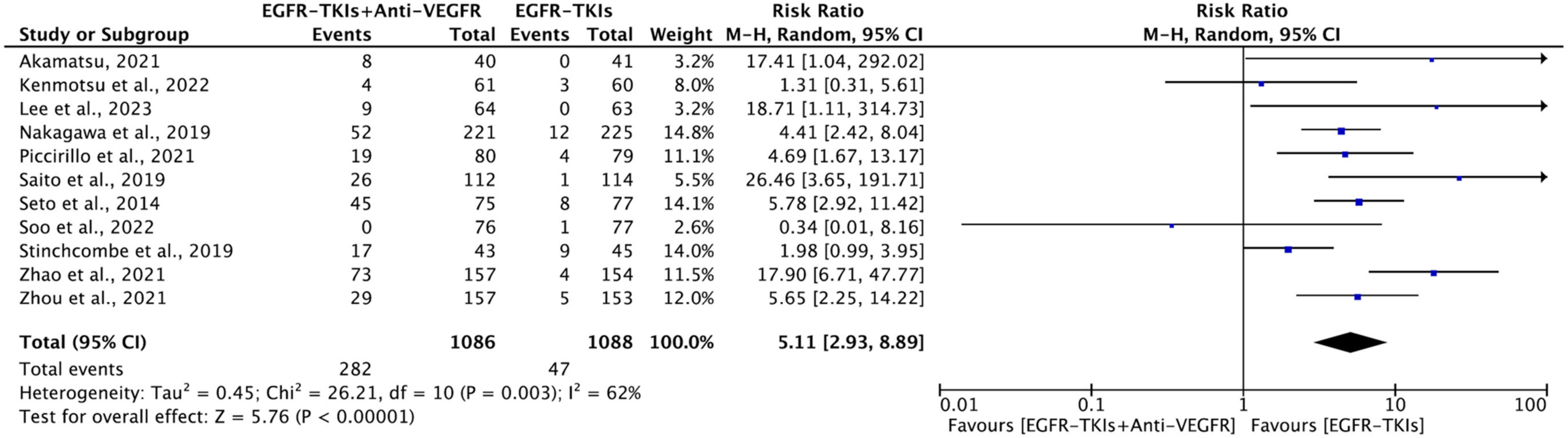

(**B**)

Study or Subgroup	EGFR-TKIs+Anti-VEGFR Events	EGFR-TKIs+Anti-VEGFR Total	EGFR-TKIs Events	EGFR-TKIs Total	Weight	Risk Ratio M-H, Fixed, 95% CI
Akamatsu, 2021	1	40	1	41	0.7%	1.02 [0.07, 15.83]
Kenmotsu et al., 2022	0	61	1	60	1.1%	0.33 [0.01, 7.89]
Lee et al., 2023	11	64	3	63	2.3%	3.61 [1.06, 12.33]
Nakagawa et al., 2019	2	221	5	225	3.7%	0.41 [0.08, 2.08]
Piccirillo et al., 2021	73	80	70	79	53.2%	1.03 [0.93, 1.14]
Saito et al., 2019	23	112	24	114	18.0%	0.98 [0.59, 1.62]
Seto et al., 2014	19	75	15	77	11.2%	1.30 [0.72, 2.36]
Stinchcombe et al., 2019	11	43	7	45	5.2%	1.64 [0.70, 3.85]
Zhao et al., 2021	6	157	1	154	0.8%	5.89 [0.72, 48.32]
Zhou et al., 2021	8	157	5	153	3.8%	1.56 [0.52, 4.66]
**Total (95% CI)**		**1010**		**1011**	**100.0%**	**1.17 [0.99, 1.37]**
Total events	154		132			

Heterogeneity: $Chi^2 = 14.76$, $df = 9$ ($P = 0.10$); $I^2 = 39\%$
Test for overall effect: $Z = 1.89$ ($P = 0.06$)

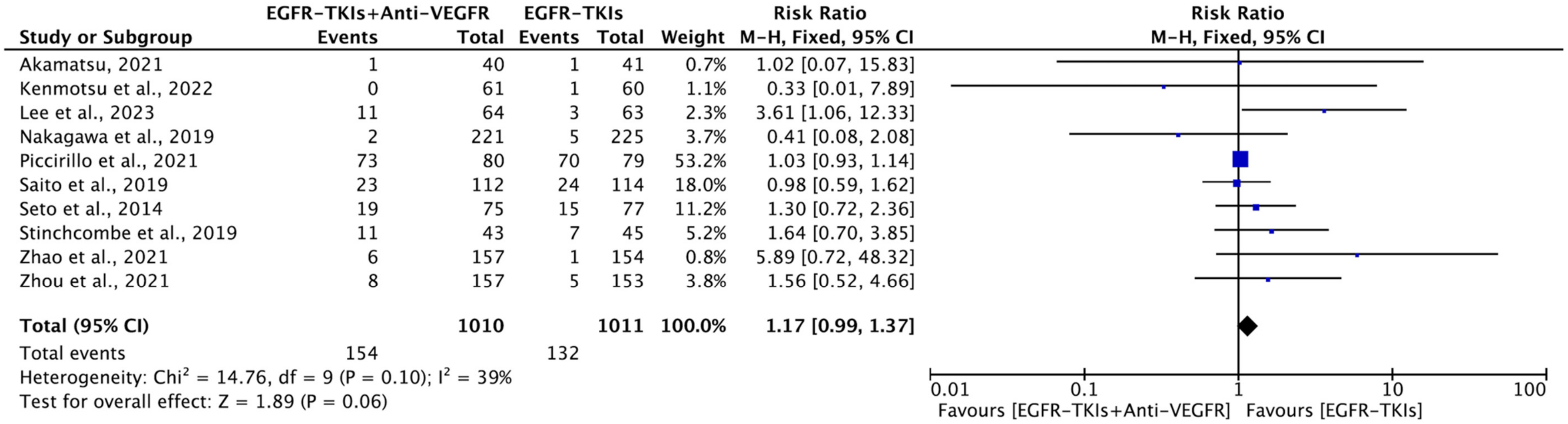

(**C**)

Study or Subgroup	EGFR-TKIs+Anti-VEGFR Events	EGFR-TKIs+Anti-VEGFR Total	EGFR-TKIs Events	EGFR-TKIs Total	Weight	Risk Ratio M-H, Fixed, 95% CI
Akamatsu, 2021	9	40	0	41	6.6%	19.46 [1.17, 323.63]
Kenmotsu et al., 2022	2	61	0	60	6.7%	4.92 [0.24, 100.37]
Lee et al., 2023	5	64	0	63	6.7%	10.83 [0.61, 191.87]
Nakagawa et al., 2019	6	221	0	225	6.6%	13.23 [0.75, 233.52]
Piccirillo et al., 2021	5	80	1	79	13.4%	4.94 [0.59, 41.32]
Saito et al., 2019	8	112	1	114	13.2%	8.14 [1.04, 64.04]
Seto et al., 2014	6	75	0	77	6.6%	13.34 [0.76, 232.75]
Soo et al., 2022	3	76	1	77	13.3%	3.04 [0.32, 28.58]
Stinchcombe et al., 2019	5	43	0	45	6.5%	11.50 [0.66, 201.90]
Zhao et al., 2021	28	157	1	154	13.5%	27.46 [3.78, 199.37]
Zhou et al., 2021	11	157	0	153	6.8%	22.42 [1.33, 377.12]
**Total (95% CI)**		**1086**		**1088**	**100.0%**	**12.22 [5.83, 25.60]**
Total events	88		4			

Heterogeneity: $Chi^2 = 3.62$, $df = 10$ ($P = 0.96$); $I^2 = 0\%$
Test for overall effect: $Z = 6.63$ ($P < 0.00001$)

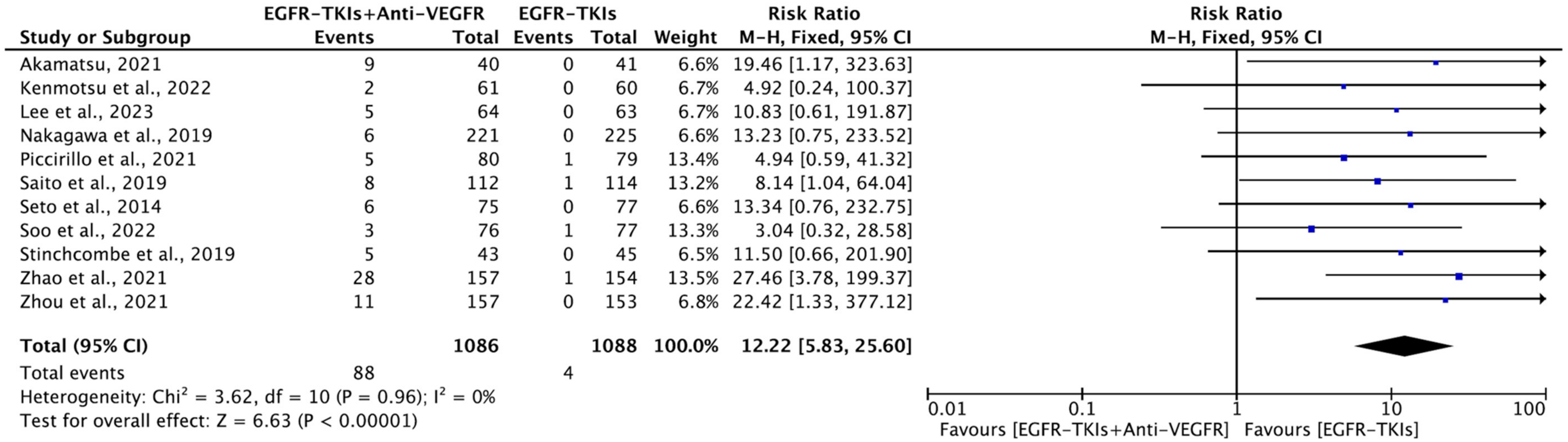

(**D**)

Study or Subgroup	EGFR−TKIs+Anti−VEGFR Events	EGFR−TKIs+Anti−VEGFR Total	EGFR−TKIs Events	EGFR−TKIs Total	Weight	Risk Ratio M−H, Fixed, 95% CI
Akamatsu, 2021	1	40	2	41	7.6%	0.51 [0.05, 5.43]
Kenmotsu et al., 2022	0	61	1	60	5.9%	0.33 [0.01, 7.89]
Lee et al., 2023	4	64	3	63	11.7%	1.31 [0.31, 5.63]
Nakagawa et al., 2019	16	221	3	225	11.5%	5.43 [1.60, 18.37]
Piccirillo et al., 2021	4	80	3	79	11.7%	1.32 [0.30, 5.69]
Saito et al., 2019	6	112	2	114	7.7%	3.05 [0.63, 14.81]
Seto et al., 2014	1	75	1	77	3.8%	1.03 [0.07, 16.12]
Soo et al., 2022	3	76	2	77	7.7%	1.52 [0.26, 8.84]
Stinchcombe et al., 2019	4	43	6	45	22.7%	0.70 [0.21, 2.30]
Zhao et al., 2021	14	157	2	154	7.8%	6.87 [1.59, 29.71]
Zhou et al., 2021	4	157	0	153	2.0%	8.77 [0.48, 161.56]
**Total (95% CI)**		**1086**		**1088**	**100.0%**	**2.25 [1.43, 3.54]**
Total events	57		25			

Heterogeneity: $Chi^2 = 13.36$, df = 10 (P = 0.20); $I^2 = 25\%$

Test for overall effect: Z = 3.49 (P = 0.0005)

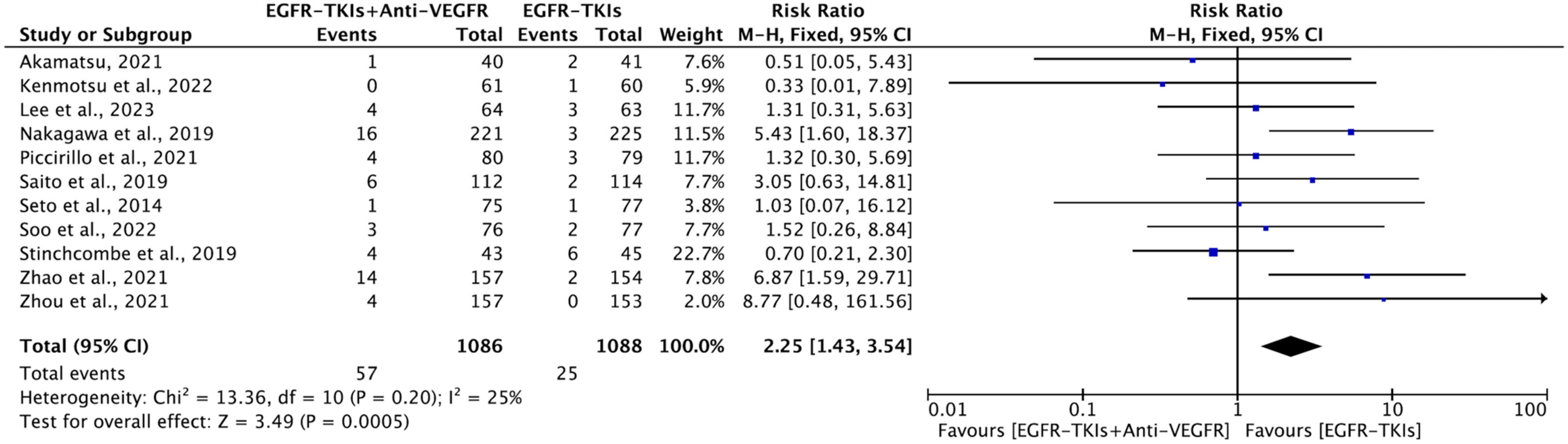

(**E**)

Supplimentary Figure S2: (A). Risk ratio of grade 3 and higher AEs between EFGR-TKIs + Anti-VEGFR and EGFR-TKIs: random-effects model [19–22,24,27,29,32–34,38]. (B). Risk ratio of hypertension between EFGR-TKIs + Anti-VEGFR and EGFR-TKIs: random-effects model [19–22,24,27,29,32–34,38]. (C). Risk ratio of skin rash between EFGR-TKIs + Anti-VEGFR and EGFR-TKIs: fixed-effects model [19–22,24,29,32–34,38]. (D). Risk ratio of proteinuria between EFGR-TKIs + Anti-VEGFR and EGFR-TKIs: fixed-effects model [19–22,24,27,29,32–34,38]. (E). Risk ratio of diarhhea between EFGR-TKIs + Anti-VEGFR and EGFR-TKIs: fixed-effects model [19–22,24,27,29,32–34,38].

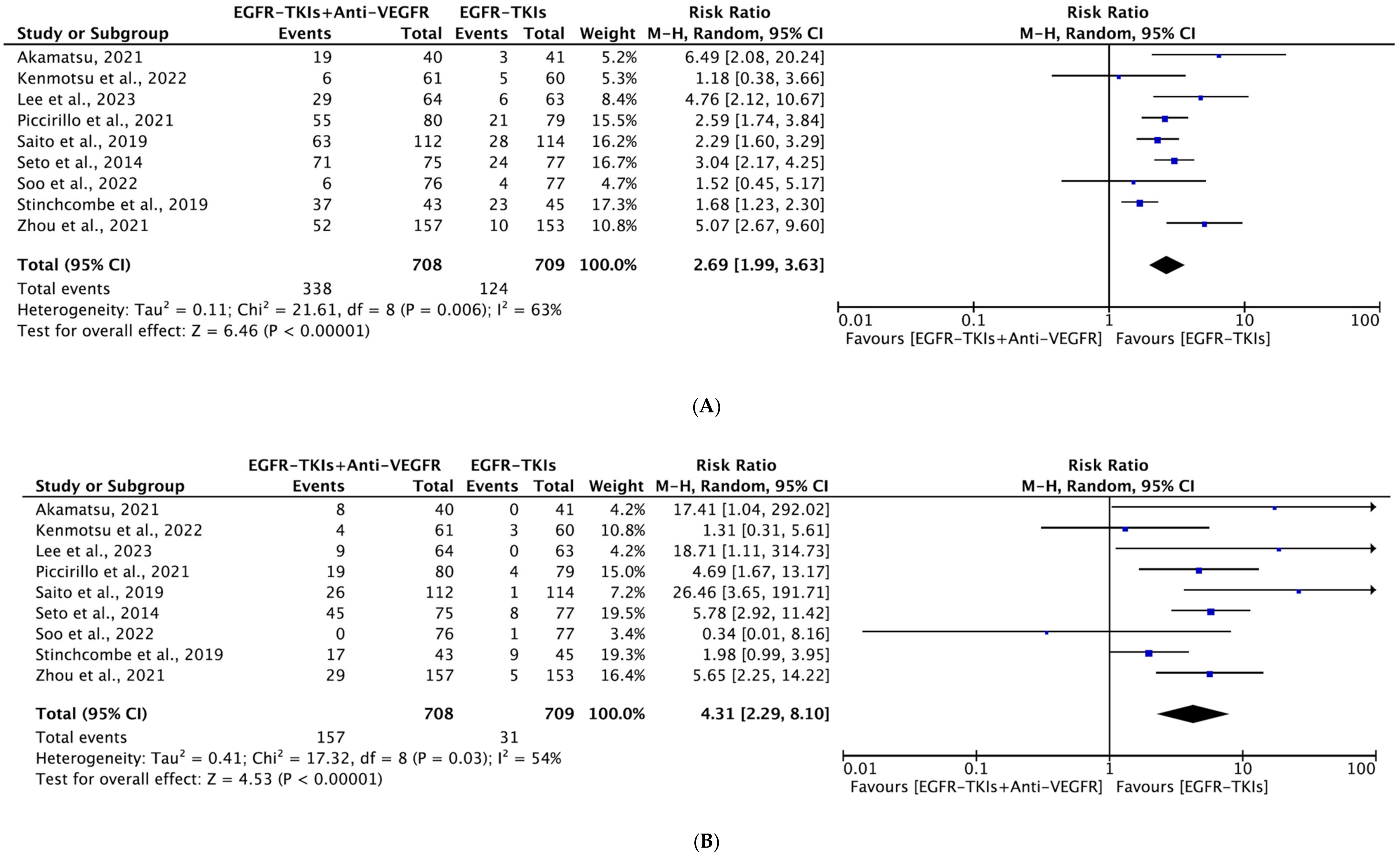
EGFR−TKIs+Anti−VEGFR
EGFR−TKIs
Risk Ratio
Study or Subgroup
Events
Total
Events
Total
Weight
M−H, Random, 95% CI
Risk Ratio
M−H, Random, 95% CI
Akamatsu, 2021 19 40 3 41 5.2% 6.49 [2.08, 20.24]
Kenmotsu et al., 2022 6 61 5 60 5.3% 1.18 [0.38, 3.66]
Lee et al., 2023 29 64 6 63 8.4% 4.76 [2.12, 10.67]
Piccirillo et al., 2021 55 80 21 79 15.5% 2.59 [1.74, 3.84]
Saito et al., 2019 63 112 28 114 16.2% 2.29 [1.60, 3.29]
Seto et al., 2014 71 75 24 77 16.7% 3.04 [2.17, 4.25]
Soo et al., 2022 6 76 4 77 4.7% 1.52 [0.45, 5.17]
Stinchcombe et al., 2019 37 43 23 45 17.3% 1.68 [1.23, 2.30]
Zhou et al., 2021 52 157 10 153 10.8% 5.07 [2.67, 9.60]
Total (95% CI) 708 709 100.0% 2.69 [1.99, 3.63]
Total events 338 124
Heterogeneity: Tau² = 0.11; Chi² = 21.61, df = 8 (P = 0.006); I² = 63%
Test for overall effect: Z = 6.46 (P < 0.00001)
0.01 0.1 1 10 100
Favours [EGFR−TKIs+Anti−VEGFR] Favours [EGFR−TKIs]
(A)
EGFR−TKIs+Anti−VEGFR
EGFR−TKIs
Risk Ratio
Study or Subgroup
Events
Total
Events
Total
Weight
M−H, Random, 95% CI
Risk Ratio
M−H, Random, 95% CI
Akamatsu, 2021 8 40 0 41 4.2% 17.41 [1.04, 292.02]
Kenmotsu et al., 2022 4 61 3 60 10.8% 1.31 [0.31, 5.61]
Lee et al., 2023 9 64 0 63 4.2% 18.71 [1.11, 314.73]
Piccirillo et al., 2021 19 80 4 79 15.0% 4.69 [1.67, 13.17]
Saito et al., 2019 26 112 1 114 7.2% 26.46 [3.65, 191.71]
Seto et al., 2014 45 75 8 77 19.5% 5.78 [2.92, 11.42]
Soo et al., 2022 0 76 1 77 3.4% 0.34 [0.01, 8.16]
Stinchcombe et al., 2019 17 43 9 45 19.3% 1.98 [0.99, 3.95]
Zhou et al., 2021 29 157 5 153 16.4% 5.65 [2.25, 14.22]
Total (95% CI) 708 709 100.0% 4.31 [2.29, 8.10]
Total events 157 31
Heterogeneity: Tau² = 0.41; Chi² = 17.32, df = 8 (P = 0.03); I² = 54%
Test for overall effect: Z = 4.53 (P < 0.00001)
0.01 0.1 1 10 100
Favours [EGFR−TKIs+Anti−VEGFR] Favours [EGFR−TKIs]
(B)

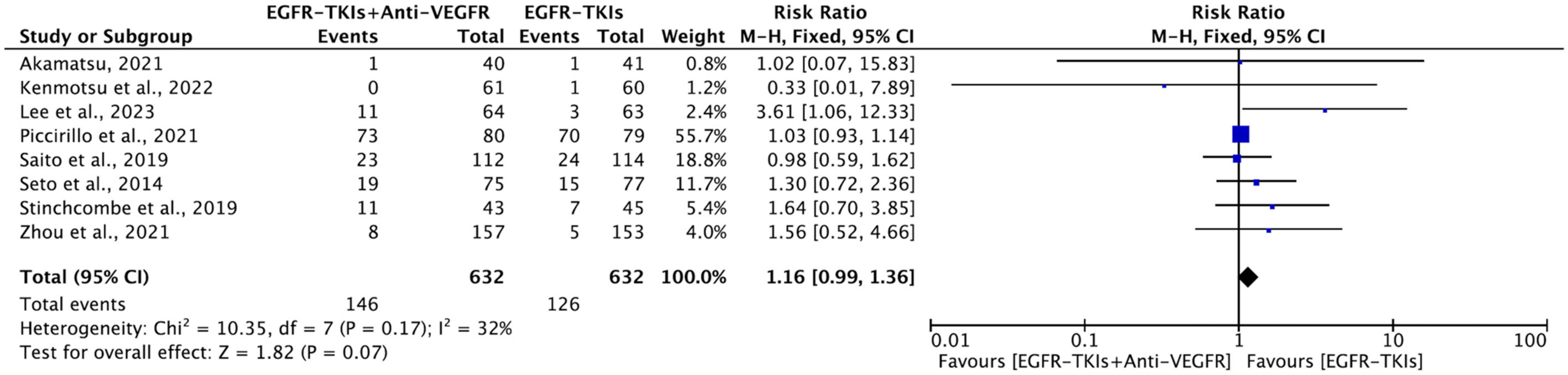
EGFR−TKIs+Anti−VEGFR
EGFR−TKIs
Risk Ratio
Risk Ratio
Study or Subgroup
Events
Total
Events
Total
Weight
M−H, Fixed, 95% CI
M−H, Fixed, 95% CI
Akamatsu, 2021 1 40 1 41 0.8% 1.02 [0.07, 15.83]
Kenmotsu et al., 2022 0 61 1 60 1.2% 0.33 [0.01, 7.89]
Lee et al., 2023 11 64 3 63 2.4% 3.61 [1.06, 12.33]
Piccirillo et al., 2021 73 80 70 79 55.7% 1.03 [0.93, 1.14]
Saito et al., 2019 23 112 24 114 18.8% 0.98 [0.59, 1.62]
Seto et al., 2014 19 75 15 77 11.7% 1.30 [0.72, 2.36]
Stinchcombe et al., 2019 11 43 7 45 5.4% 1.64 [0.70, 3.85]
Zhou et al., 2021 8 157 5 153 4.0% 1.56 [0.52, 4.66]
Total (95% CI) 632 632 100.0% 1.16 [0.99, 1.36]
Total events 146 126
Heterogeneity: Chi² = 10.35, df = 7 (P = 0.17); I² = 32%
Test for overall effect: Z = 1.82 (P = 0.07)
0.01
0.1
1
10
100
Favours [EGFR−TKIs+Anti−VEGFR]
Favours [EGFR−TKIs]

(**C**)

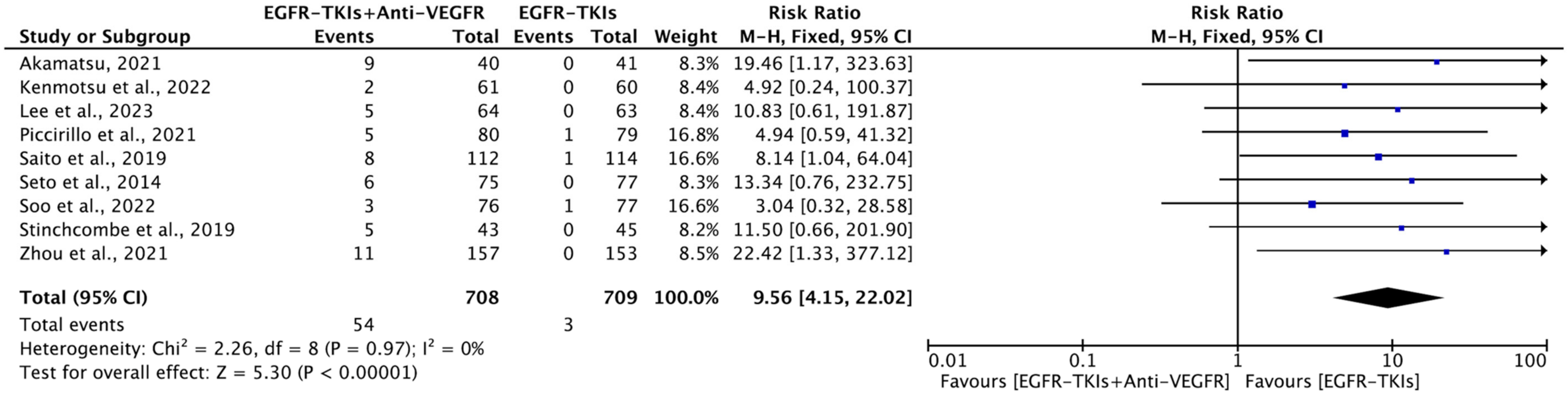
EGFR−TKIs+Anti−VEGFR
EGFR−TKIs
Risk Ratio
Risk Ratio
Study or Subgroup
Events
Total
Events
Total
Weight
M−H, Fixed, 95% CI
M−H, Fixed, 95% CI
Akamatsu, 2021 9 40 0 41 8.3% 19.46 [1.17, 323.63]
Kenmotsu et al., 2022 2 61 0 60 8.4% 4.92 [0.24, 100.37]
Lee et al., 2023 5 64 0 63 8.4% 10.83 [0.61, 191.87]
Piccirillo et al., 2021 5 80 1 79 16.8% 4.94 [0.59, 41.32]
Saito et al., 2019 8 112 1 114 16.6% 8.14 [1.04, 64.04]
Seto et al., 2014 6 75 0 77 8.3% 13.34 [0.76, 232.75]
Soo et al., 2022 3 76 1 77 16.6% 3.04 [0.32, 28.58]
Stinchcombe et al., 2019 5 43 0 45 8.2% 11.50 [0.66, 201.90]
Zhou et al., 2021 11 157 0 153 8.5% 22.42 [1.33, 377.12]
Total (95% CI) 708 709 100.0% 9.56 [4.15, 22.02]
Total events 54 3
Heterogeneity: Chi² = 2.26, df = 8 (P = 0.97); I² = 0%
Test for overall effect: Z = 5.30 (P < 0.00001)
0.01
0.1
1
10
100
Favours [EGFR−TKIs+Anti−VEGFR]
Favours [EGFR−TKIs]

(**D**)

Study or Subgroup	EGFR−TKIs+Anti−VEGFR Events	Total	EGFR−TKIs Events	Total	Weight	Risk Ratio M−H, Fixed, 95% CI
Akamatsu, 2021	1	40	2	41	9.5%	0.51 [0.05, 5.43]
Kenmotsu et al., 2022	0	61	1	60	7.3%	0.33 [0.01, 7.89]
Lee et al., 2023	4	64	3	63	14.5%	1.31 [0.31, 5.63]
Piccirillo et al., 2021	4	80	3	79	14.5%	1.32 [0.30, 5.69]
Saito et al., 2019	6	112	2	114	9.5%	3.05 [0.63, 14.81]
Seto et al., 2014	1	75	1	77	4.7%	1.03 [0.07, 16.12]
Soo et al., 2022	3	76	2	77	9.5%	1.52 [0.26, 8.84]
Stinchcombe et al., 2019	4	43	6	45	28.1%	0.70 [0.21, 2.30]
Zhou et al., 2021	4	157	0	153	2.4%	8.77 [0.48, 161.56]
**Total (95% CI)**		**708**		**709**	**100.0%**	**1.35 [0.77, 2.34]**
Total events	27		20			

Heterogeneity: $Chi^2 = 5.24$, df = 8 (P = 0.73); $I^2 = 0\%$
Test for overall effect: Z = 1.05 (P = 0.29)

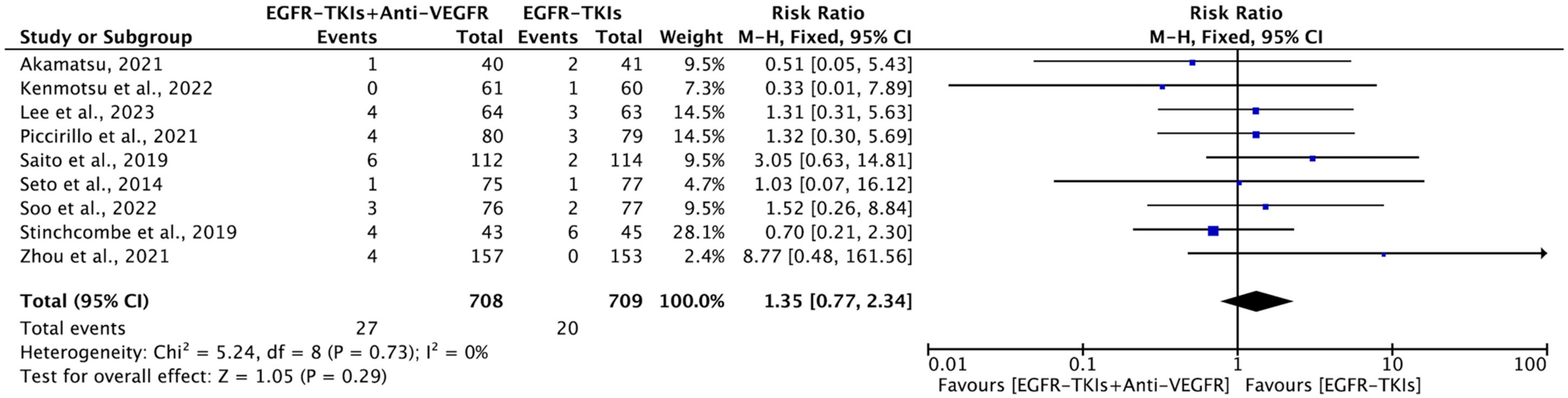

(**E**)

Supplementary Figure S3: (A). Risk ratio of grade 3 and higher AEs between EFGR-TKIs plus Bevacizumab and EGFR-TKIs: random-effects model [19,20,22,24,27,29,33,34,38]. (B). Risk ratio of hypertension between EFGR-TKIs plus Bevacizumab and EGFR-TKIs: random-effects model [19,20,22,24,27,29,33,34,38]. (C). Risk ratio of skin rash between EFGR-TKIs plus Bevacizumab and EGFR-TKIs: fixed-effects model [19,20,22,24,29,33,34,38]. (D). Risk ratio of proteinuria between EFGR-TKIs plus Bevacizumab and EGFR-TKIs: fixed-effects model [19,20,22,24,27,29,33,34,38]. (E). Risk ratio of dirhhea between EFGR-TKIs plus Bevacizumab and EGFR-TKIs: fixed-effects model [19,20,22,24,27,29,33,34,38].

Supplimentary Table S1. Adverse events in patients receiving EGFR-TKIs plus Anti-VEGFR-combination and EGFR-TKI alone.

Author, year	N (Comb)	N (Mono)	AE G3 Comb	AE G3 Mono	Rash - Comb	Rash - Mono	HTN - Comb	HTN - Mono	Proteinuria - Comb	Proteinuria - Mono	Diarrhea - Comb	Diarrhea - Mono
Akamatsu, 2021 [38]	40	41	19 (48%)	3 (7%)	1 (3%)	1 (2%)	8 (20%)	0 (0%)	9 (23%)	0 (0%)	1 (3%)	2 (5%)
Kenmotsu et al., 2022 [33]	61	60	6 (10%)	5 (8%)	0 (0%)	1 (1.7%)	4(6.6%)	3 (5%)	2 (3.3%)	0 (0%)	0 (0%)	1 (1.7%)
Nakagawa et al., 2019 [32]	221	225	76 (34%)	20 (9%)	2 (19%)	5 (2%)	52 (24%)	12 (5%)	6 (3%)	0 (0%)	16 (7%)	3 (1%)
Piccirillo et al., 2022 [29]	80	79	55 (69%)	21 (27%)	73 (92%)	70 (88%)	19 (24%)	4 (5%)	5 (6%)	1 (1%)	4 (5%)	3 (4%)
Saito et al., 2019 [34]	112	114	63 (56%)	28 (25%)	23 (21%)	24 (21%)	26 (23%)	1 (1%)	8 (7%)	1 (1%)	6 (5%)	2 (2%)
Seto et al., 2014 [22]	75	77	71 (95%)	24 (31%)	19 (25%)	15 (19%)	45 (60%)	8 (10%)	6 (8%)	0 (0%)	1 (1%)	1 (1%)
Soo et al., 2022 [27]	76	77	6 (8%)	4 (5%)	0 (0%)	0 (0%)	0 (0%)	1 (1%)	3 (4%)	1 (1%)	3 (3%)	2 (3%)
Stinchcombe et al., 2019 [24]	43	45	37 (86%)	23 (51%)	11 (26%)	7 (16%)	17 (40%)	9 (20%)	5 (12%)	0 (0%)	4 (9%)	6 (13%)
Zhao et al., 2021 [21]	157	154	123 (78%)	10 (6%)	6 (3.8%)	1 (0.6%)	73 (47%)	4 (2.6%)	28 (18%)	1 (1%)	14 (9%)	2 (1%)
Zhou et al., 2021 [20]	157	153	52 (33%)	10 (7%)	8 (5%)	5 (3%)	29 (19%)	5 (3%)	11 (7%)	0 (0%)	4 (3%)	0 (0%)
Lee et al., 2023 [19]	64	63	29 (45%)	6 (10%)	11 (17.2%)	3 (4.8%)	9 (14.1%)	0 (0%)	5 (7.8%)	0 (0%)	4 (6.3%)	3 (4.8%)

EFGR-TKIs= Epidermal Growth Factor Receptor Tyrosine Kinase Inhibitors; Anti-VEGFR-TKIs: Anti-Vascular Endothelial Growth Factor Receptor Tyrosine Kinase Inhibitors; Mono= Monotherapy; AEs: Adverse Events; Comb= Combination Therapy; AE G3 = Adverse Drug Events Grade 3 and higher; Rash – Skin rash; HTN = Hypertension

Supplimentary Table S2. Relative risk of adverse events in patients with advanced NSCLC treated with EGFR-TKIs plus Bevacizumab combination compared to EGFR-TKIs alone.

	EFGR-TKIs + Beva	EGFR-TKIs			Heterogeneity	
**Adverse events**	**Event/Total**	**Event/Total**	**RR (95%CI)**	**p-value**	I^2	**p-value**
Grade 3 AEs	338/708 (49.4%)	124/709 (14.2%)	2.69 (1.99, 3.63)	<0.00001	63%	0.006
Skin rash	146/632 (15.2%)	126/632 (13.1%)	1.16 (0.99, 1.36)	0.07	32%	0.17
Hypertension	157/708 (25.9%)	31/709 (4.3%)	4.31 (2.29, 8.10)	<0.00001	54%	0.03
Diarrhea	27/708 (5.2%)	20/709 (2.3%)	1.35 (0.77, 2.34)	0.29	0%	0.73
Proteinuria	54/708 (8.1%)	3/709 (0.4%)	9.56 (4.15, 22.02)	<0.00001	0%	0.97

NSCLC: Non Small Cell Lung Cancer; EFGR-TKIs= Epidermal Growth Factor Receptor Tyrosine Kinase Inhibitors; AEs: Adverse Events; Beva: Bevacizumab; RR: Relative Risk; CI: Confidence Interval

Supplimentary Table S3. Demographic chrecteristics of the patients in studies that were a part of systematic review (qualitative synthesis) in additions to ones that were included in the meta-analyses.

Global = US/Canada/Other; Asia = Japan/China; Europe (Italy); EFGR-TKI= Epidermal Growth Factor Receptor Tyrosine Kinase Inhibitor; VEGFR=Vascular Endothelial Growth Factor Receptor; ITT= Intention to Treat Analysis; EGFR= Epidermal Growth Factor Receptor; + = Yes

Author, year	EGFR-TKI	Anti-VEGFR agent	Phase	Study Region	Trial name	EGFR mutation	Line of Treatment	Prior Treatment	Patients (N)	Age (yrs.) (Median)	Female, (%)	% Asian	ITT analysis
Groen et al., 2013 [37]	Erlotinib	Sunitinib	II	Global		Undefined	Second-line	Cytotoxic	132	60	36	2	
Herbst et al., 2011 [36]	Erlotinib	Bevacizumab	III	Global	BeTa	Undefined	Second-line	Cytotoxic	636	65	46	6	+
Natale et al., 2009 [31]	Gefitinib	Vandetanib	II	Global		Undefined	Second-line	Cytotoxic	168	62	40		+
Natale et al., 2011 [30]	Erlotinib	Vandetanib	III	Global	NCT00364351	Undefined	Second-line	Cytotoxic	1240	61	38	33	+
Scaggliotti et al., 2012 [28]	Erlotinib	Sunitinib	III	Global		Undefined	Second-line	Cytotoxic	960	61	39	11	+
Spigel et al., 2011 [26]	Erlotinib	Sorafenib	II	US		Undefined	Second-line	Cytotoxic	166	65	47	0	
Spigel et al., 2018 [25]	Erlotinib	Pazopanib	II	US		Undefined	Second-line	Cytotoxic	192	67	46		
